# Food and social media: a research stream analysis

**DOI:** 10.1007/s11301-023-00330-y

**Published:** 2023-02-18

**Authors:** Ruth Areli García-León, Thorsten Teichert

**Affiliations:** Chair of Marketing and Innovation, University of Hamburg, Von-Melle-Park 5, 20146 Hamburg, Germany

**Keywords:** Food, Social-media, Word-of-mouth, Social-network-analysis, Text-mining, e-WOM, M00

## Abstract

Interest in food and online communication is growing fast among marketing and business scholars. Nevertheless, this interest has been not exclusive to these areas. Researchers from different disciplines have focused their research on different concepts, target populations, approaches, methodologies, and theoretical backgrounds, making this growing body of knowledge richer, but at the same time difficult to analyze. In order to have a broader overview of this topic, this study analyzes the existent literature regarding food and social media in social sciences in order to identify the main research streams and themes explored. With this purpose, the present paper uses bibliometric methods to analyze 1356 journal articles by means of factor and social network analysis. The study contributes by revealing 4 clusters containing 11 dominant research streams within the social sciences, determining the linkages among the main research discourses, and recommending new future topics of research.

**Supplementary Information:**

The online version contains supplementary material available at 10.1007/s11301-023-00330-y.

## Introduction

Food and social media is highly a controversial topic. While some studies point out that the use of social media can be associated with an increase of unhealthy food intake and Body Mass Index (BMI) (Coates et al. 2019a; Khajeheian et al. 2018), other studies, as well as the OECD and the American Heart Association suggest that the use of social media could be used to sensitize the population regarding obesity and to promote public health regarding food (Chau et al. 2018; Li et al. 2013; OECD 2017).

People use the World Wide Web and social media to seek and share information, for social interaction, and to be part of a social network (Kavanaugh et al. 2005; Whiting and Williams 2013). Billions of opinions are shared on social networks every day (Mostafa 2019), breaking barriers across geographical distance and bringing people closer (Rimjhim et al. 2020). Social networks and online communities facilitate consumer-to-consumer communication (Sloan et al. 2015), and influence consumers’ opinions, attitudes, consumption experiences, brand perceptions, purchasing decisions, as well as post-purchase communication and evaluation, among others (Jansen et al. 2009; Mangold and Faulds 2009; Teichert et al. 2020).

The rapid growth of online communication among consumers has increased academic interest in electronic word of mouth (e-WOM). Zinko et al. (2021) define e-WOM as the “web-mediated exchange of information which occurs when one person tells another about their experience with a service or product” (p. 526). E-WOM includes blogs, online reviews, ratings, messages posted on online groups, and social media posts (Hennig-Thurau and Walsh 2003). Either as a topic of consumer health, sustainability, or as an opportunity for management development, studies regarding food and social media are gaining importance. Scholars from different disciplines have used different approaches, methodologies, theoretical backgrounds, and populations targets to address this topic. Additionally, due to the novelty of some internet-based communication tools, and the rapid emergence of additional ones, new concepts, definitions, and approaches are emerging too, making this growing body of knowledge difficult to explore.

Although the scope of food and social media research has partly been disclosed in literature reviews, these focus on a particular sub-segment of food consumption, a specific target population, area of research, research method, or a specific new technology or social media. For example, Chau et al. (2018) centered their research on the role of social media in nutrition interventions for adolescents and young adults. Rounsefell et al. (2020) explored the impact of social media exposure to image-content on body image and food choices in young adults. Chapman et al. (2014) analyzed literature regarding the use of social media for public health communication in order to explore the potential of social media as a tool to combat foodborne illness. De Veirman et al. (2019) studied the persuasive power of social media influencers over young children. Dute et al. (2016) examined literature regarding the promotion of physical activity, healthy nutrition, and overweight prevention among adolescents and students, through mobile apps. Allman-Farinelli and Gemming (2017) explored the state of the art in dietary assessment, using smartphone and digital technology regarding technology mediated interventions for dietary change. Tao et al. (2020) studied the use of text mining as a big data analysis tool for food science and nutrition. And Ventura et al. (2021) analyzed the topic of food in social media from a consumer-oriented point of view. However, there are no studies offering a general overview of a broad sample of articles within the social sciences regarding food and the use of social media.

Given this, the aim of this paper is to provide a broad bibliometric review for marketing and business scholars, companies, and organizations on past and current research regarding food and social media within the social sciences, in order to reveal the main addressed topics, as well as for suggesting future topics of research in this field of knowledge. To achieve the results, this research uses the co-word analysis of Keywords. Co-word analysis (Callon et al. 1983) is a type of bibliometric method which seeks to find connections among concepts that co-occurs in document abstracts, titles, or keywords as assessed by the authors (Zupic and Čater 2015). By conducting a co-word analysis of keywords, the present study aims to reveal the main research streams regarding food and social media studied in the social sciences. First, statistical analyses are applied to identify research streams as well as their interconnections in an objective manner. Single research streams are then analyzed in detail by a manual inspection of their key publications. Focal issues of past and current research are highlighted and opportunities for future research are identified.

## Methodology

### Co-word analysis

One of the most used bibliometric methods is co-citation analysis. Nevertheless, while co-citation analysis connects documents, authors, or journals in order to find the intellectual structure, the knowledge base, or influences on a research field (Small 1977; Zupic and Čater 2015) the co-word analysis uses the actual words contained in documents to determine relationships among concepts that represent a conceptual space of a field (Zupic and Čater 2015). In co-citation analysis, it is assumed that the more two items are cited together, the more likely is that their content is related, and since it takes time to accumulate citations, the analysis reflects the state of the field in the past and not how it could look now or tomorrow (Zupic and Čater 2015). In this regard, the co-word analysis offers a more actual state of the field since authors choose the words, concepts, titles, and keywords that best represent their studies. In their articles, authors construct different realities linking scientific and technical concepts that are shared by a specific research community (Callon et al. 1983). Therefore, the co-word analysis is more content-driven than the co-citation analysis.

The main target of this analysis is the keywords contained in the articles since keywords are chosen by the authors because they represent in a few words, the main content of the study. Web of Science database (WoS) is frequently used for bibliometric studies in management and organization, and it contains different valuable bibliographical data for indexed documents that include title, article type, authors, keywords, keywords plus, abstract and subject categories or areas, among others (Zupic and Čater 2015). Besides the Author Keywords, WoS provides Keywords Plus. They are index terms automatically generated from the titles of cited articles in an article that augment traditional keyword retrieval (Clarivate 2020). Therefore, this research analyzes the Author Keywords and the Keywords Plus provided by WoS.

### Identification of literature

**Fig. 1:**
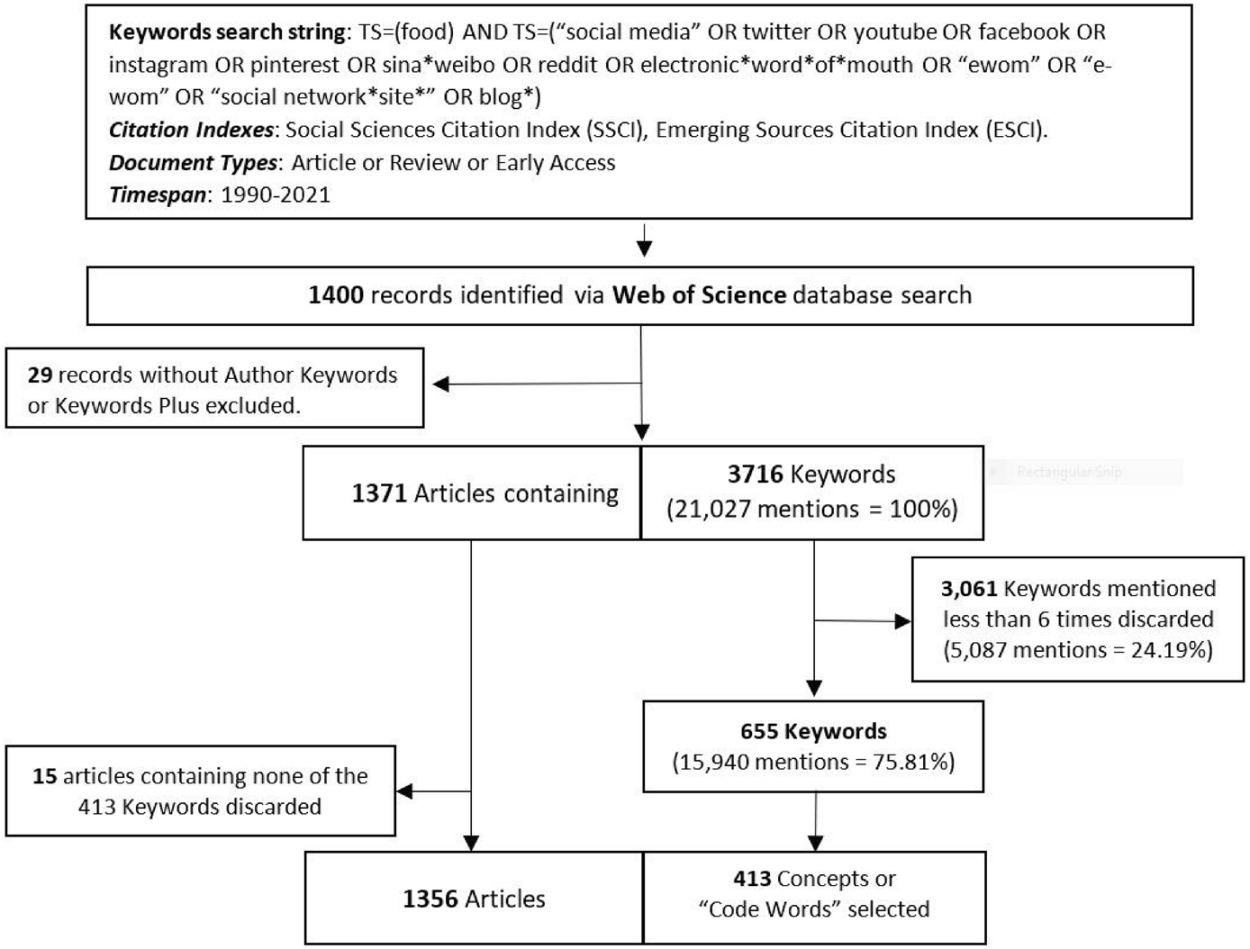
Sample generation process by steps

The search of documents was made on WoS by using a Keywords string containing the main concepts related to the objective of the research (see Fig. 1 for the overall design, search string, and interim steps taken). Although most of the well-known social media such as Youtube or Twitter appeared in the 2000s, some authors consider that the development of social media started during the 80 s with the introduction of USENET, a type of internet discussion system, real-time online chat services such as Compu Serve’s CB Simulator (1980), the Internet Relay Chat (IRC) (1988), or AOL’s chat rooms (1989) (Edosomwan et al. 2011; Lake 2009; Sajithra and Patil 2013). Others establish this development in the 90 s when the World Wide Web became public and web blogs, list-servers, and e-mail services allowed users to form online communities exploding networked communication (Simonova et al. 2021; van Dijck 2013). Therefore, in order to have a broader number of articles and consequently a broader scope regarding food and social media research in Social Sciences, the timespan 1990 to 2021 and the citation indexes *Social Sciences Citation Index (SSCI)* and *Emerging Sources Citation Index (ESCI)* were used as limiters. The ESCI extends the scope of publications of WoS by including around 3,000 peer-reviewed publications that although they are not yet recognized internationally, meet the WoS high-quality criteria (Francis 2021). Besides, *Articles, Reviews, or Early Access* articles were included in order to capture the most recent published works. Early Access articles in WoS Core Collection are fully indexed articles that the publisher makes available online in a nearly final state (e.g. Articles in Press, Published Ahead of Print, Online First, etc.), they lack publication date, volume, issue, and page number (Clarivate 2021).

With this information, an initial database of 1400 records was created on July, 20 of 2021. Nevertheless, only articles containing Author Keywords and/or Keywords Plus were included; therefore, 29 articles without author Keywords and Keywords Plus were removed. In the end, just 1371 were included in the next analysis.

A first analysis of Keywords contained in the 1371 articles was made by using the KHCoder, a text-mining and text-analysis application (https://khcoder.net/en/). To avoid the analysis of joined words separately, a total of 31 words strings, also called Force Pick Up Words, were chosen to extract different words as one concept (e.g. qualitative_research, corporate_social_responsibility) (see Table S1 in Supplementary material). The word frequency list revealed a total of 3,716 keywords and a total of 21,027 mentions. In order to include just the most representative concepts in the analysis, just concepts mentioned more than 5 times were included. Hence, just 655 Keywords representing 75.81% of all mentions were included in the second analysis.

The second step was an analysis of concepts, conducted by both researchers, in order to find similarities among words due to meaning, writing differences, use of abbreviations, or use of signs to unite words.

After this analysis, a list of 413 Keywords or “code words” containing the initial 655 Keywords was generated (the complete list of words and code words (*) could be seen in Table S2 in Supplementary material). This list of code words was introduced to KHCoder in order to generate a crosstab containing the concepts included in every article. As a result, 15 articles containing none of the 413 Keywords were discarded for further analysis.

### Data analysis

The data were analyzed by using the package UCINET 6 (Borgatti et al. 2002), one of the most used software for network visualization (Zupic and Čater 2015), in order to generate an overall concept co-occurrence matrix. By executing a core-periphery analysis the core keywords contained in the food and social media literature were separated from the periphery keywords. The stable solution was found in 50 iterations (fitness = 0.609).

Then, a factor analysis was conducted using SPSS in order to group keywords based on their co-occurrences. Factor analysis can determine which indicators, in this case, keywords, may be grouped together. Factor analysis is known as a data reduction technique (Sallis et al. 2021). In order to identify groups of bibliometric data, researchers have used different statistical techniques such as factor analysis, cluster analysis, multidimensional scaling, or multivariate analysis (Chen et al. 2016; Leydesdorff and Welbers 2011; Ravikumar et al. 2015; Wang et al. 2012; Yang et al. 2012), although, for practical use, some authors have not found a difference between cluster analysis and factor analysis (Lee and Jeong 2008).

The use of factor analysis has a long tradition in co-word analysis. Considered a quantitative form of content analysis, it can substitute commonly practiced techniques for content analysis, providing precision and validity in the resulting categories while investing less time and resources (Leydesdorff and Welbers 2011; Simon and Xenos 2004). Many studies have used factor analysis in co-word analysis as a reliable method to discover linkages among scientific documents. For example, by using the words contained in the titles and abstracts of research articles, Leydesdroff (1989) used factor analysis and cluster analysis to find linkages among biochemistry documents. Leydesdorff and Hellsten (2005) studied words related to stem-cell by using factor analysis. Leydesdorff and Zhou (2008) used factor analysis to analyze words of journal titles using Chinese characters. Wang et al. (2014) analyzed keywords from core journals in the field of domestic knowledge discovery by using factor and cluster analysis. Yan et al. (2015) analyzed the intellectual structure of the field of the Internet of Things by means of factor and cluster analysis of keywords. Gan and Wang (2015) used factor analysis to map the intellectual structure of social media research in china by using keywords, and Sun and Teichert (2022) used factor analysis to study the research landscape of ‘scarcity’ by using author keywords.

In the specific application field of bibliometrics, the method identifies different research streams (Kuntner and Teichert 2016). By reducing the number of variables in a dataset, the factor analysis finds patterns and therefore, the underlying structure of the data (Wendler and Gröttrup 2016). There are different methods to extract factors. This study applied a principal component analysis (PCA) with an orthogonal factor rotation Varimax with Kaiser Normalization of 15 iterations. Varimax is a very popular rotation method in which each factor represents a small number of variables and each variable tends to be associated with one or a small number of factors (Abdi 2003). It enhances clarity, interpretability, and efficiency when distinguishing among the extracted factors (Simon and Xenos 2004). PCA finds the linear combination between indicators that extract the most variance in the data and uses both common and specific variance to extract a solution (Sallis et al. 2021). Therefore, in order to find the main research streams regarding food and social media, the number of variables (i.e. Keywords) was reduced to identify the underlying structure based on the overall variance. By performing factor analysis, determined keywords are assigned to determined factors based on their factor loadings. Factor loads (FL) inform about the representativeness of a determined keyword for a determined factor, and the usage of a keyword in a research stream (Kuntner and Teichert 2016; Sun and Teichert 2022). That means that the keywords assigned to one factor are more likely to co-occur than the keywords of other factors. Therefore, by using this method, factors were interpreted as single research streams.

As a result of the analysis, 12 factors emerged, which explain 51.175% of the total variance (see Table S3 in Supplementary material for the complete concepts per factor). Factor 11 was found to address issues related to the pharmaceutical industry and the Food and Drug Administration of United States (FDA) guidance documents. This factor was omitted in the further analysis, as it primarily addresses the pharmaceutical industry does not have a direct relationship with food and social media.

In order to further identify group similarities across research streams, a cluster analysis in SPSS was conducted. Cluster analysis finds natural groups present in the data, but hidden, by identifying important and defining properties (Sallis et al. 2021). This analysis revealed four main research clusters that the researchers named: Psychological Research Realm, Action-Oriented Research, Broader Communication Issues, and Service Industry Discourse (see Table 12 for a summary of research clusters and their characteristics).

## Results and discussion

In the following, the four different clusters of research are explained in detail considering the most representative publications of every factor or research stream.

### Psychological research realm

The Psychological Research Realm contains four research streams; therefore, it is the biggest of the four clusters. These research clusters address mainly, the impact of social media use on consumers. It includes the streams “online tools for healthy diet intervention programs,” “food and use of apps,” “online food advertising exposure,” and “social media and mental disorders.”

#### Research stream on “online tools for healthy diet intervention programs” (Factor 1)

The first research stream explains 18.94% of the variance of keyword relationships, indicating a research stream of first-highest distinction. While *obesity* and *diet* were the most often listed keywords (130 and 123 mentions), the research stream was best represented (in terms of factor loadings) by the keywords *diet* (FL = 0.922)*,* followed by *intervention. Program,* related to *(physical) activity, nutrition, prevention, adult, overweight, and association* constitute the remainders of the top ten keywords. An inspection of the remaining 103 keywords confirms this focus on application-oriented topics from the perspective of healthy diet interventions. Thus, this research stream clearly addresses the topic “use of online tools for healthy diet intervention programs.”

Representative publications of this research stream (see Table 1) reference each more than 14 keywords of factor 1. Regarding theories and conceptualizations, most of the articles refer to healthy diets and the use of online tools. Thus, an inclusive and shared research discourse can be diagnosed.

**Table 1:** Top ten factor 1 keywords and representative publications addressing online tools for healthy diet intervention programs

Factor 1. Variance explained: 18.94%		
Keyword	# of mentions	FL
Diet	123	0.922
Intervention	70	0.908
Physical_activity	73	0.889
Obesity	130	0.877
Nutrition	106	0.865
Prevention	47	0.841
Adult	61	0.833
Overweight	42	0.824
Program	24	0.799
Association	43	0.792
Other 103 Keywords* with FL ≥ 0.738		

Representative publications	# referenced keywords
Tobey et al. (2019) Nutrients	19
Chau et al. (2018) Int. J. Med. Inform	19
Helle et al. (2017) BMC Public Health	16
Ahmad et al. (2020) Nutrients	16
Gupta et al. (2019) Indian Pediatrics	16
Røed et al. (2019) BMC Public Health	15
Dumas et al. (2020) J. Acad. Nutr. Diet	15

*Further keywords (sorted by decreasing FL): fruit; eating; weight-loss; healthy; weight; behavior_change; survey; young_adult; trial; child; control; vegetable; weight-gain; low-income; feeding; parent; mother; lifestyle; habit; consumption; risk-factor; intake; disease; school; randomized_controlled-trial; ehealth; pattern; adolescent; public_health; peer; self-efficacy; status; practice; infant; nutrition_education; choice; gender; meal; population; qualitative_research; social_marketing; environment; prevalence; pregnancy; validation; metaanalysis; fast-food; energy; united-state; american; cooking; nutritional; family; index; recipe; sugar; support; loss; group; exercise; education; taste; body-mass; adherence; barrier; cancer; student; emerge; young; efficacy; promotion; policy; recommendation; healthcare; home; life; qualitative; time; trend; drinking; research; validity; diabetes; outcome; focus; assessment; cognitive; store; design; implementation; inequality; study; stigma; method; awareness; people; vegan_vegetarian; evaluation; factor; process; availability; age; bias; label; disparity; natural; individual; photography; university

A closer look at these articles (selected by maximum number of referenced keywords) provides insights about the methods used, the online tools evaluated, as well as the types of insights gained from this research discourse (Table 1 right columns). These articles address the use of online tools for healthy diet intervention programs by using randomized and controlled trial groups, among others. The studies analyze the development of novel online tools as well as the efficacy of other healthy diet intervention tools.

The consumption of junk foods, fast foods, sugar-sweetened beverages, and carbonated drinks and beverages is associated with higher body mass index in children and adolescents due to their high content of free sugar and energy (Gupta et al. 2019). In order to promote public health sensitizing the population regarding obesity, the use of social media and new technologies has been recommended by the OECD and the American Heart Association (Li et al. 2013; OECD 2017).

In this regard, this research stream contains protocols of novel internet-based intervention tools to promote healthy diets (Helle et al. 2017; Røed et al. 2019), as well evaluations about the effectivity of online tools for intervention programs, and for the delivery of healthy eating information and recipes, among others. Ahmad et al. (2020) evaluated the effect of the family-based intervention program (REDUCE) on children’s eating behaviors and dietary intake via face-to-face and social media by using Facebook and a WhatsApp group to deliver information about the intervention and as platforms of interaction and problem solving. The authors found small changes in consumption of unhealthy snacks, as well as fruits and vegetables, without clinical impact. Dumas et al. (2020) explored the effects of an evidence-informed healthy eating blog written by a registered dietitian, finding no effects on dietary intakes, food-related behaviors, and body weight.

While these former studies did not reveal a strong positive impact, there are other studies showing positive results. For example, with the aim of evaluating the value of social media for delivering healthy diet interventions, Chau et al. (2018) found that the majority of the studies associated with this topic, from 2006 to 2016, showed positive outcomes regarding the use of only basic social media features. Tobey et al. (2019) evaluated the success of the Food Hero marketing campaign and suggest that in order to disseminate recipes to low-income audiences through social marketing campaigns, is recommended to understand the target audience, to add healthy/customizable recipes to family “go-to” recipe rotations considering the generational influences on family meals, and to create websites that meet the target audience criteria (e.g. simple and visually interesting).

By delivering healthy diet interventions through social media or online tools, studies in this research stream targeted mainly parents. Future research might evaluate the efficacy of social media or novel online tools by targeting parents and children separately, and by delivering strategies designed for each group.

#### Research stream on “online food advertising exposure” (Factor 5)

Explaining 2.78% of the variance of keyword relationships, the fifth research stream indicates a research stream of fifth-highest distinction. Here, the most often mentioned keywords were *marketing* and *advertising* (82 and 63 mentions). However, in terms of factor loadings, the research stream was best represented by the keywords *advertising* (FL = 0.915)*,* followed by *marketing. Exposure* related to *(unhealthy) food, television, advergame, beverage, celebrity, youtube,* and *endorsement* constitute the remainders of the top ten keywords. The inspection of the remaining 14 keywords confirms the online advertising exposure approach. Thus, this research stream clearly addresses the topic “online food advertising exposure.”

Representative publications (see Table 2), selected by the highest number of reference keywords, reference each more than 6 keywords of factor 5, and address the concept of *influencer marketing*, and among other social media, they analyze mainly YouTube videos, sharing an inclusive research discourse.

**Table 2:** Top ten factor 5 keywords and representative publications addressing online food advertising exposure

Factor 5. Variance explained: 2.78%		
Keyword	# of mentions	FL
Advertising	63	0.915
Marketing	82	0.857
Unhealthy_food	14	0.829
Television	32	0.815
Exposure	45	0.81
Advergame	10	0.797
Beverage	22	0.755
Celebrity	17	0.757
Youtube	28	0.755
Endorsement	14	0.737
Other 14 Keywords* with FL ≥ 0.697		

Representative publications	# referenced keywords
Coates et al. (2019b) Front. Psychol	13
Coates et al. (2020) Int. J. Environ. Res. Public Health	10
De Veirman et al. (2019) Front. Psychol	8
Coates et al. (2019a) Pediatr. Obes	8
Kent et al. (2019) Pediatr. Obes	8
Coates et al. (2019c) Pediatrics	7

*Further keywords (sorted by decreasing FL): influencer; cue; literacy; alcohol; digital; response; preference; australium; identification; smoking; self-management; popularity; power; campaign

A closer look at these articles reveals that four of six articles of this research stream were led by the same author. In general, the articles of this research stream address the exposure to food advertising online by means of content analysis, questionnaires, and multivariate analysis, among others.

Regarding food and beverage marketing content on social media, Kent et al. (2019) found that although children and adolescents are exposed to unhealthy food and beverage marketing on social media, adolescents were more highly exposed to food marketing than children through user‐generated, celebrity‐generated content, and other entertainment content. Regarding food and beverage products featured on YouTube videos of influencers who are popular with children, it was found that less healthy products were the most frequently featured, branded, presented in the context of eating out, described positively, not consumed, and featured as part of an explicit marketing campaign, than healthy products (Coates et al. 2019b).

Studies in this research stream have proved the persuasive power of social media influencer promotion of food, and their impact on children’s food intake, even when including a protective disclosure, due to their credibility and familiarity with children. Some authors situate social media influencers as a new type of advertising source that combines the merits of e-WOM and celebrity endorsement (De Veirman et al. 2019). YouTubers featuring videos of food and beverages high in fat, sugar, and/or salt (HFSS) are valued highly by children because they are viewed to fulfill their needs. Children develop sympathetic attitudes towards YouTubers because they are not strangers to them (Coates et al. 2020). Children look up to popular influencers who have certain celebrity status and are willing to identify with them while taking on their lifestyles, attitudes, and beliefs. Therefore, (marketing) messages spread by them are perceived as highly credible WOM, rather than as advertising, due to their perceived authenticity (i.e., they have no commercial interests) (De Veirman et al. 2019).

It has been discovered that children exposed to influencer marketing in a YouTube video of a branded unhealthy snack (with and without an advertising disclosure) consumed more of the marketed snack and significantly increased intake of unhealthy snacks specifically whereas the equivalent marketing of healthy foods had no effect. Therefore, it has been concluded that influencer marketing increases children's immediate intake of the promoted snack, even when including a “protective” advertising disclosure, which does not reduce the effect of influencer marketing (Coates et al. 2019a, 2019c). Results reveal that increasing the promotion of healthy foods on social media could not be an effective strategy to encourage healthy dietary behaviors in children (Coates et al. 2019c).

In sum, most of the articles in this research stream address children and adolescents’ exposure to unhealthy food influencer marketing contained in YouTube videos. Further research could evaluate the use of influencer marketing on children for healthy food intake, not just in YouTube, but also in other video content social media like TikTok, or Instagram. Other studies could compare different target groups (e.g. adults, adolescents, and children) in different countries.

#### Research stream on “social media and mental disorders” (Factor 8)

The eights research stream explains 1.93% of the variance of keyword relationships, indicating a research stream of eight-highest distinction. The research stream was best represented (in terms of factor loadings) by the keywords *depression* (FL = 0.793)*,* followed by *anxiety.* The same words were, as well the most listed keywords (18 and 17 mentions)*. Addiction, disorder, symptom, distress, psychological, stress, well-being,* and *personality* constitute the remainders of the top ten keywords. An inspection of the remaining 6 keywords confirms this focus on application-oriented topics from the perspective of mental disorders. Thus, this research stream clearly addresses the topic “social media and mental disorders.”

Representative publications of this research stream (see Table 3) reference each more than 4 keywords of factor 8. Regarding theories and conceptualizations, although this research stream has not a leading theory, they analyze different mental disorders and their relationship with social media. Thus, an inclusive and shared research discourse can be diagnosed.

**Table 3:** Top ten factor 8 keywords and representative publications addressing social media and mental disorders

Factor 8. Variance explained: 1.93%		
Keyword	# of mentions	FL
Depression	18	0.793
Anxiety	17	0.749
Addiction	12	0.72
Disorder	16	0.686
Symptom	9	0.638
Distress	8	0.549
Psychological	14	0.516
Stress	15	0.511
Well-being	9	0.507
Personality	16	0.505
Other 6 Keywords* with FL ≥ 0.483		

Representative publications	# referenced keywords
Panno et al. (2020) Front. Psychiatry	8
Reaves et al. (2019) Subst. Use Misuse	7
Kicali et al. (2021) Dusunen Adam	7
Charzynska et al. (2021) Addict. Behav	6
Bountress et al. (2021) Eur. J. Psychotraumatol	5
Kircaburun et al. (2021) Psychiatry Investig	5
Bogolyubova et al. (2018) SAGE Open	5

*Further keywords (sorted by decreasing FL): sensitivity; scale; use; mental; covid-19; event

A closer look at these articles (selected by maximum number referenced keywords) provides insights about the methods applied and types of insights gained from this research discourse (Table 3 right columns). These articles address social media use and mental disorders by using questionnaires, addiction scales, and personality inventories, among others. Hence, antecedents and consequences of social media use and mental disorders are analyzed.

Regarding the antecedents of addictive behaviors, it was found that personality traits and gender, as well as certain mental disorders, are associated with different behavioral addictions. For example, the profiles “elevated levels of gaming and pornography addictions” as well as “highest levels of all addictions” are predominantly male, while the profile “elevated levels of study, Facebook, shopping, and food addictions” are almost exclusively female (Charzynska et al. 2021). Besides, it was concluded that “individuals higher in anxiety sensitivity/hopelessness used food or alcohol to cope which, in turn, significantly predicted unhealthy snacking, and hazardous drinking, respectively” (Reaves et al. 2019, p. 921).

Regarding the use of social media and its impact on mental disorders, Kicali et al. (2021) found that although food addiction is associated with some personality traits, personal habits, and psychiatric symptoms, more than five hours a day of social media consumption hat a direct relationship with internet and eating addiction. Kircaburun et al. (2021) found that a Problematic YouTube Use (PYU), which refers to different activities like watching specific YouTube channels or viewing online video games, is associated with loneliness and depression. Other works in this research stream explored images shared on social media and their relationship with mental disorders. E.g., Bogolyubova et al. (2018) concluded that while in Russian language people shared more images of food with hashtags for stress, images of alcohol were associated with stress hashtags, and hashtags for fear were related to the “scary” in popular culture and not to psychological distress.

Other works in this research stream addressed the impact of the COVID-19 Pandemic on mental health. Bountress et al. (2021) determined that instead of a single overarching COVID-19 impact, there are discrete impacts of various COVID-related factors. Therefore, they suggest a five-factor COVID model (i.e. exposure, worry, housing/food instability, social media, substance use) which is able to predict the risk of mental health symptomology, as well as other adverse sequelae of the COVID-19 pandemic at large. On the other hand, Panno et al. (2020) confirmed that COVID-19 related distress is associated with alcohol problems, social media, and food addiction symptoms. Following this line of research, future research might explore further the use of social media for mental health.

#### Research stream on “food and the use of apps” (Factor 12)

The twelfth research stream explains 1.32% of the variance of keyword relationships, and is the research stream of twelfth-highest distinction. *Mobile* and *adoption,* were the most often listed keywords (24 mentions each). Nevertheless, the research stream was best represented (in terms of factor loadings) by the keywords *application* (FL = 0.621)*,* followed by *mobile.* The remainders of the top five words were *(Smart)phone* and *app.* A closer look at the main keywords confirms its orientation to application-oriented topics from the perspective of the use of apps, focusing clearly on the topic “food and the use of apps.”

Representative publications reference each more than 2 keywords of factor 12 (see Table 4). Although this research stream has not a leading theory, most of the articles investigate the topic of food and the use of apps, sharing an inclusive research discourse. The representative publications chosen by the highest number of referenced keywords (Table 4 right columns), address the use of apps in relation to food by means of literature review, questionnaires, and interviews, mainly. Among others, social media content, as well as antecedents, and contingencies regarding food tourism are analyzed.

**Table 4:** Top ten factor 12 keywords and representative publications addressing food and the use of apps

Factor 12. Variance explained: 1.32%		
Keyword	# of mentions	FL
Application	11	0.621
Mobile	24	0.619
(Smart) phone	20	0.556
App	15	0.515
Adoption	24	0.358

Representative publications	# referenced keywords
Quamar et al. (2020) Disabil. Rehabil.-Assist. Technol	5
Allman-Farinelli and Gemming (2017) Curr. Diabetes Rep	4
Shah et al. (2020) Asia Pac. J. Market. Logist	3
Alalwan (2020) Int. J. Inf. Manage	3
Paas et al. (2021) Int. J. Market Res	3
Tonkin et al. (2017) JMIR mHealth uHealth	3
Feijoo-Fernandez et al. (2020) Prof. Inf	3
Williams et al. (2020) Automat. Softw. Eng	3
Dute et al. (2016) JMIR mHealth uHealth	3

Information Communication Technology (ICT) (e.g. internet; mobile technology; and social media platforms among others) influence the daily living activities of persons, specifically Instrumental Activities of Daily Living (IADL) (e.g. activities requiring complex problem solving, cognitive function, coordination, and scheduling) (Quamar et al. 2020). In this regard, children interact with and consume visual advertising when visiting sites or applications related to online gaming (23%), food and distribution (18%), entertainment (8%) and fashion (8%), and when using smartphones with Internet access, Chilean children receive 14 min per hour of use of visual advertising more than from other media, such as television (Feijoo-Fernandez et al. 2020).

Regarding the antecedents of the use of mobile phones and apps for service purposes, it was found that the adoption of services and apps is driven by individual’s mobile phone technology maturity and business development (Paas et al. 2021). An analysis of user’s feedback on Twitter of four prominent food delivery apps and app store reviews of these apps revealed that the main concerns of users are related to issues regarding customer service, orders, food, delivery, time, app, money, drivers, and restaurants (Williams et al. 2020). Regarding mobile dining (e.g. use smartphone apps, to find restaurants, to read food menus, to select food, and to order it) it was found that consumers’ purchase intention is shaped by perceived values (i.e. navigation system, review valence, credibility, as well as service, and food quality) (Shah et al. 2020).

Other studies explored the use of smartphone apps for healthy lifestyles and dietary change. While Allman-Farinelli and Gemming (2017) concluded that apps have proven to be effective for glycemic control but not yet regarding weight loss and food intake, other studies found that monitoring apps enable users to set targets and monitor themselves. Besides, it is possible to acquire tailored feedback, and subsequently to raise awareness and increase motivation regarding dietary intake and physical activity. Moreover, apps with incorporated social features, characterized as social media, facilitate social interaction and support, can provide social comparison and social support (Dute et al. 2016). Concerning the development of smartphone apps to reduce sugar-sweetened beverage consumption among disadvantaged young adults in nonurban settings or indigenous communities, Tonkin et al. (2017) identified the importance of design to facilitate comprehension, and that in order to increase satisfaction the use of social features such as audio, leader boards, games, and team challenges could be helpful.

Studies in this research stream explored the use of specific apps for service purposes or dietary change, in just one region or sample. Further research could conduct comparative studies among apps, with different target groups in different geographical areas or regions.

### Action-oriented research

This research cluster analyzes the content of social media and its impact on consumers' food risk information seeking and perception, behavioral intention and buying of green products online, as well as food tourism for destination image and its promotion. It includes the research streams “online food risk communication,” “behavioral intention and buying online,” and “social media and food tourism.”

#### Research stream on “online food risk communication” (Factor 3)

This research stream of third-highest distinction explains 3.79% of the variance of keyword relationships. *Communication* and *risk* were the most often listed keywords accounting 151 and 102 mentions respectively. However, in terms of factor loading, it was best represented by the keywords (*food) safety* (FL = 0.827)*,* followed by (*risk) communication.* The remainders of the top ten keywords were the keywords *public* and *(risk) perception* related to *safety, (food) risk, crisis,* and *amplification*. The remaining 35 keywords indicate its focus on themes from the perspective of online communication, addressing clearly the topic “online food risk communication.”

Table 5 displays the representative publications of this research stream, which reference each more than 8 keywords of factor 3. Most of them address the risk communication concept, sharing therefore an inclusive research discourse. These articles address the topics of online media consumption and food risk by means of surveys and quantitative content analysis, among others. They focus mainly on the coverage of topics related to health risk, consumers´ food risk information seeking, and consumers´ risk perception.

**Table 5:** Top ten factor 3 keywords and representative publications addressing online food risk communication

Factor 3. Variance explained: 3.79%		
Keyword	# of mentions	FL
Food_safety	50	0.827
Risk_communication	26	0.788
Crisis	40	0.787
Amplification	10	0.76
Communication	151	0.73
Risk_perception	17	0.721
Public	40	0.709
Risk	102	0.704
Safety	31	0.689
Food_risk	9	0.673
Other 35 Keywords* with FL ≥ 0.661		

Representative publications	# referenced keywords
Wu (2015) J. Bus. Res	11
Chapman et al. (2014) Perspect. Public Health	10
Shan et al. (2014) Public Underst. Sci	10
Moon and Shim (2019) J. Commun. Manag	10
Tiozzo et al. (2020) J. Med. Internet Res	9
Hanssen et al. (2018) Jcom-J. Sci. Commun	9
Niu et al. (2022) J. Risk Res	9

*Further keywords (sorted by decreasing FL): news; coverage; issue; falsehood; credibility; modify; trust; source; monitoring; media; science; benefit; knowledge; fear; perception; internet; traditional; mass; reputation; governance; genetically; technology; frame; framework; emotion; seek; infection; gratification; illness; web; message; foodborne; perspective; allergy; tool

Some studies in this research stream explore how online information sources cover different healthy risk themes. For example, during the 2008 Irish dioxin contamination of food, Shan et al. (2014) found that social media responded faster than traditional media, using offline and online media news messages as primary sources, in reporting limited topics. Related to the coverage of biological, chemical, nutritional food risks, and related safety issues, Tiozzo et al. (2020) discovered that the most widely covered topics were nutritional risks and news about outbreaks, controls, and alerts. Moreover, national sources covered food risks, especially during food emergencies whereas thematic sources devoted major attention to nutritional topics.

In regard to the antecedents of consumers’ online information seeking behavior, concerning food safety issues, Wu (2015) concluded that Facebook use intention is determined by risk perception, emotion, social trust, and support. Regarding Genetic Modification (GM) issues, (Hanssen et al. 2018) discovered that the frequency with which people seek information is low, and it is driven by a positive attitude toward science and technology, trust in organizations, negative trust in regulations, as well as by gender and educational level. As a tool for food safety risk, specifically, to combat foodborne illness, Chapman et al. (2014) identified that the use of social media could be helpful for public health and food safety risk, since social media provide access to real people´s discussions and feedback, allow communicators to reach people where they are, create communities, and can be used to build credibility by providing decision-making evidence.

Regarding risk perception, some studies in this research stream found that risk perception depends on the topics and the online source used by consumers. For example, mixed media have a stronger positive relationship regarding public risk perception (PRP), than traditional media or internet social media (Niu et al. 2022). And, in the case of bovine spongiform encephalopathy (BSE), individuals exposed to more internet news had higher risk perceptions in terms of how BSE could affect themselves, while respondents exposed to social networking sites were concerned about how the disease could affect others (Moon and Shim 2019).

With most of the articles of this research stream addressing risk perception, or consumers’ food risk information seeking, further research could explore how social media could be used effectively for public health and food safety risk by using quantitative and qualitative methods of research.

#### Research stream on “behavioral intention and buying online” (Factor 4)

The fourth research stream explains 3.02% of the variance of keyword relationships, indicating a research stream of fourth-highest distinction. The research stream was best represented (in terms of factor loadings) by the keywords *organic* (FL = 0.765)*,* followed by *purchase,* although *attitude* and *intention* were the most often listed keywords (79 and 66 mentions)*. Theory* and *(planed) behavior* related to *buying, food-intake*, *belief,* and *acceptance,* were the remainders of the top ten keywords. As it can be confirmed by analyzing the remaining 20 keywords, the focus of this research stream relies on the perspective of behavioral intention, addressing thus the topic of “behavioral intention and buying online.”

Representative publications of this research stream (see Table 6), selected by the highest number of referenced keywords, contain each more than 7 keywords of factor 4. Addressing the Theory of Planned Behavior (TPB) and/or the Theory of Reasoned Action (TRA) (Ajzen and Fishbein 1980), most of the articles address the concept of “behavioral intention” regarding green, or organic products, showing an inclusive and shared research discourse.

**Table 6:** Top ten factor 4 keywords and representative publications addressing behavioral intention and buying online

Factor 4. Variance explained: 3.02%		
Keyword	# of mentions	FL
Organic	36	0.765
Purchase	38	0.757
Planned_behavior	15	0.755
Buying	15	0.722
Attitude	79	0.707
Intention	66	0.687
Food-intake	18	0.647
Belief	23	0.646
Acceptance	28	0.634
Theory	59	0.622
Other 20 Keywords* with FL ≥ 0.586		

Representative publications	# referenced keywords
Jaini et al. (2019) Int. J. Pharm. Healthc. Mark	10
Li and Jaharuddin (2021) Cogent Bus. Manag	9
Matharu et al. (2021) Manag. Environ. Qual	9
Tariq et al. (2019) Asia Pac. J. Market. Logist	9
Pop et al. (2020) Information	9
Bukhari et al. (2020) Sustainability	8
Lim and Lee-Won (2017) Telemat. Inform	8

*Further keywords (sorted by decreasing FL): consumer_behavior; green; determinant; equation; structural; norm; social; commerce; willingness; fit; behavioral; concern; china; meat; opinion; influence; decision-making; sharing; personal; action

With six of seven articles using TPB or TRA, this research stream addresses the topic of behavioral intention regarding green products by means of structural equation modeling.

The TPB is an improved version or extension of the Theory of Reasoned Action (TRA) (Ajzen 1991; Hofmeister-Tóth et al. 2011). The TPB differs from the TRA, “in that it takes into account perceived as well as actual control over the behavior under consideration” (Ajzen 1985, p. 12). Ajzen (1985) explains that actions are controlled by intentions. Therefore, the TPB is a model that predicts behavior based on the *intention to perform the behavior* and the *perceived behavioral control* where the *attitude towards the behavior*, *the subjective norm,* and the *perceived behavioral control* influence intention (Aertsens et al. 2009).

Studies of this research stream concluded that the information contained in social media tools can influence the intention to perform a behavior regarding green or organic products. Considering green cosmetics purchase intentions, Pop et al. (2020) point out that social media can increase consumers’ environmental concerns, consumers’ attitudes, subjective norms, altruistic and egoistic motivations, and therefore consumers’ green cosmetics purchase intentions. By using the value-belief-norm theory and the elaboration likelihood model, Jaini et al. (2019) discovered that e-WOM communications influences consumers’ green cosmetics purchase decisions, with personal norm affecting this choice, especially when they are actively involved in obtaining positive feedback via e-WOM communication. In addition, pro-environmental beliefs, which eventually affect consumers’ personal norms, are affected positively by hedonic, and altruistic value.

Regarding organic food, it was confirmed that consumers’ attitudes towards organic food can be shaped by social media forums and informative webpages featuring product quality and certification. They have a great moderating effect on purchase ratings and reviews that positively influence consumers’ online impulse buying behavior (Tariq et al. 2019). Background factors like information (i.e., social media information and labeling), individual (i.e., health consciousness and purchase attitude), and social (i.e., self-perceived vegetarian and environmentalism), impact consumers’ intention of purchasing organic food (Li and Jaharuddin 2021). Lim and Lee-Won (2017) discovered that dialogic retweets (i.e. retweeting user mentions addressed to an organization), are more persuasive than monologic tweets because dialogic retweets lead to a higher level of subjective norms, more favorable attitudes toward behavior, and greater intention to adopt the behavior advocated by an organic food organization in the messages. On the other hand, a lifestyle of health and sustainability influences the attitude of customers toward sustainable consumption and therefore, consumers’ sustainable consumption behavior (Matharu et al. 2021). Furthermore, regarding western imported food products in a Muslim country, Bukhari et al. (2020) found that product attributes, price, self-concept, brand trust, personality, and religiosity are positively correlated with consumers’ purchase intention in Pakistan.

This research stream concluded that the information contained in social media can influence the intention to consume green or organic products. Nevertheless, it is known that there is an intention-behavior gap, identified between positive attitudes toward organic products and actual purchase behavior (Padel and Foster 2005; Pearson et al. 2011). Thus, further research could explore, by means of mixed methods, how social media could reduce the intention-behavior gap.

#### Research stream on “social media and food tourism” (Factor 10)

The tenth research stream explains 1.54% of the variance of keyword relationships, indicating a research stream of tenth-highest distinction. While *image* (58 mentions) and *destination, (content) analysis and instagram* (30 mentions each) were the most often listed keywords, the research stream was best represented (in terms of factor loadings) by the keywords *destination* (FL = 0.645)*,* followed by *authenticity. Place,* related *to travel, culinary, image, wine,* and *gastronomy* constitute the remainders of the top ten keywords. These 10 keywords in this research stream confirm the application-oriented topics from the perspective of food tourism. Therefore, this research stream clearly addresses the topic “social media and food tourism.”

Representative publications of this research stream (see Table 7) reference each more than 2 keywords of factor 10. Regarding theories and conceptualizations, although this research stream has not a leading theory, they analyze food tourism and its relationship with social media. Thus, an inclusive and shared research discourse can be determined.

**Table 7:** Top ten factor 10 keywords and representative publications addressing social media and food tourism

Factor 10. Variance explained: 1.54%		
Keyword	# of mentions	FL
Destination	30	0.645
Authenticity	10	0.64
Place	18	0.586
Culinary	12	0.583
Image	58	0.562
Travel	16	0.525
Wine	12	0.504
Content_analysis	30	0.486
Gastronomy	9	0.467
Instagram	30	0.318

Representative publications	# referenced keywords
Bachman et al. (2021) Tour. Anal	6
Filieri et al. (2021) Tourism Manage	5
Yu and Sun (2019) Tourism Manage	5
Ramirez-Gutierrez et al. (2021) Tour. Recreat. Res	5
Li et al. (2020) Sustainability	4
Demirkol and Cifci (2020) Eur. J. Tour. Res	3
Okumus (2021) Tour. Rev	3
Vrontis et al. (2021) J. Place Manag. Dev	3
Tiggemann und Zaccardo (2018) J. Health Psychol	3

A closer look at these articles (selected by maximum number of referenced keywords) provides insights about the methods applied and types of insights gained from this research discourse (Table 7 right columns). These articles address food tourism related to social media use by means of content analysis, semi-structured interviews, and literature review, among others. The articles analyzed social media content, as well as antecedents and contingencies regarding social media and food tourism.

The use of social media to increase destination image or to promote a food destination is the main focus of this research stream. Over the past two decades, the key themes regarding food tourism were authenticity through food experiences, the offer of unique food experiences, food tourism and sustainability, as well as the use of food destination in marketing; nevertheless, Okumus (2021) suggests that future studies should focus on the role of social media in promoting food tourism experiences, among others. In this regard, Filieri et al. (2021) found that on Instagram, users communicate their destination brand love through photographs of some destination attributes (e.g. people, food, weather, etc.) accompanied by specific positive emotions (e.g. attractiveness, pleasure, amazement, etc.) or providing emotional support during a destination crisis. Besides, Ramirez-Gutierrez et al. (2021) concluded that in TripAdvisor, tourists’ communications of gastronomic experiences contain both aesthetic and personal values.

Other studies in this research stream reveal social media strategies and how specific online tools can help to promote food destinations. While memories influence positively the loyalty for a food destination (Bachman et al. 2021), the description of food on TikTok brings an effect of intention to travel and to obtain information, impacting the affective image of a destination and increasing potential tourists’ attention (Li et al. 2020). As a tool to advertise food-based cities, Yu and Sun (2019) recommend the use of Instagram to attract the attention of consumers including hashtags to reach more users and to generate interactivity. Moreover, the endorsement of celebrity chefs on social media can help to promote cities as culinary destinations by giving provocativeness (i.e. attractiveness and customer engagement), credibility (i.e. trustworthiness, leading, and reliability), and supportiveness (i.e. localism and match-up) (Demirkol and Cifci 2020). Besides, Vrontis et al. (2021) suggest that the support interactions between destination managers and stakeholders by using online technology; can be transformed into a word-of-mouth source that could affect perceptions and sustainable development of the territory producing the place brand.

Finally, by conducting a content analysis of 600 Instagram images containing the hashtag #fitspiration, Tiggemann and Zaccardo (2018) found that most images of women contained objectifying elements, and only one body type: thin and toned. Authors point out that although ‘fitspiration’ images may be inspirational for viewers, they contain elements that could affect negatively the viewer’s body image.

This research stream analyzed the role of social media in food tourism on Instagram, TikTok, and Tripadvisor. Further research might explore the use of further social media tools in order to enrich this research stream with comparisons among tools and countries.

### Broader communication issues

This research cluster analyses online communications regarding Alternative Food Networks (AFN), online communication, and eating disorders, as well as the analysis of online food related data by means of novel tools. This cluster includes the research streams “sustainable food communication online,” “analysis of online food related data,” and “online communication and eating disorders.”

#### Research stream on “sustainable food communication online” (Factor 6)

Explaining the 2.66% of the variance of keyword relationships, this research stream of sixth-highest distinction was best represented (in terms of factor loadings) by the keyword *sustainability* (FL = 0.727)*,* followed by *agriculture,* although *network* and *sustainability* were the most often listed keywords (68 and 60 mentions)*.* The remainders of the top ten keywords, were the words *innovation*, *system, economy, chain, alternative, supply,* and *farmer*. The remaining 24 keywords confirm the focus on sustainable food communication. Thus, this research stream clearly addresses the topic “sustainable food communication online.”

The most representative articles of this research stream (see Table 8) were selected by the highest number of keywords referenced, in this case, each more than 6 keywords of factor 5. Without a leading theory, most of the articles rely on the concept of AFN, and local food networks or systems. They address the topic of sustainable food and online communication, linked both by means of content analysis, data mining, semi-structured interviews, surveys, and participant observation, among others. Media content is investigated, as well as antecedents and contingencies regarding sustainable food communication online.

**Table 8:** Top ten factor 6 keywords and representative publications addressing sustainable food communication online

Factor 6. Variance explained: 2.66%		
Keyword	# of mentions	FL
Sustainability	60	0.727
Agriculture	33	0.695
Innovation	40	0.684
System	56	0.672
Network	68	0.666
Economy	20	0.639
Chain	24	0.636
Alternative	11	0.63
Supply	20	0.622
Farmer	19	0.6
Other 24 Keywords* with FL ≥ 0.597		

Representative publications	# referenced keywords
Bos and Owen (2016) J. Rural Stud	9
Kummer and Milestad (2020) Sustainability	9
Martindale (2021) Agric. Human Values	9
Pilar et al. (2019) Sustainability	8
Ashtab and Campbell (2021) Sustainability	8
Campos and Zapata (2017) Environ. Polit	8
Schumilas and Scott (2016) Asia Pac. Viewp	7

*Further keywords (sorted by decreasing FL): rural; climate; waste; local; space; entrepreneurship; movement; community; urban; trade; market; resource; dynamics; organization; justice; city; activism; reduction; business; access; challenge; change; citizen; social_media_marketing

Regarding the antecedents of the use of internet communications, in this research stream, it was found that initiators and participants of AFN are individual shoppers and nascent activists that organize strategies, build networks, and use internet communications to extend their reach, and expand linkages to emancipatory spaces of global and social justice movements (Schumilas and Scott 2016). Online spaces (e.g. websites and social media platforms) supplement the socio-material connections in AFNs’ offline spaces providing a ‘virtual reconnection’ or an additional real for reconnection (Bos and Owen 2016). By using social media, participants in citizen-drive initiatives (e.g. for waste-prevention) create collaborative local networks to develop green/sustainable consumption practices (Campos and Zapata 2017). Exploring communications with the hashtag #sustainability on Twitter, Pilar et al. (2019) discovered six communities (i.e. Environmental Sustainability, Sustainability Awareness, Renewable Energy and Climate Change, Innovative Technology, Green Architecture, and Food Sustainability), and 6 hashtags related to sustainability (i.e. innovation, environment, climate change, corporate social responsibility, technology, and energy).

Regarding the use of online communications by producers and intermediaries, it was found that producers establish consumers’ trust by satisfying the consumer´s desire for safe food, and that they use social media to construct food materiality and the perception of this materiality in order to fit the consumer´s ideal of freshness (Martindale 2021). Besides, Kummer and Milestad (2020) discovered that social media is used as an advertising tool in the growing practice of box schemes (i.e. a type of locally oriented distribution system used by community supported agriculture (CSA) farms or enterprises) in Europe. Other works in this research stream studied the motivations for buying sustainable agricultural products (e.g. Ashtab and Campbell 2021).

Further research could explore not just the use of social media for communication, but also how these communications influence behavior-change and sustainable food consumption among their participants.

#### Research stream on “analysis of online food related data” (Factor 7)

The seventh-highest distinction research stream explains 2.14% of the variance of keyword relationships. In terms of factor loadings, the keywords *(sentiment) analysis* (FL = 0.74)*,* and *tweet* are the main keyword representing this research stream*.* The top ten keywords were led by *twitter* with 102 mentions, followed by *(sentiment) analysis* and *datum* with 35 mentions each. *Halal, detection, topic*, *mining, classification,* and *sentiment* are the remainders of the top ten keywords. Analyzing all keywords, it can be confirmed the use of words related to methods for the analysis of online data. Therefore, this research stream addresses the topic of “analysis of online food related data.”

Although the representative publications (see Table 9), with more than 5 keywords of factor 7, do not share a leading theory, they share a research discourse by analyzing Twitter communications. With three articles led by the same author, articles in this research stream address the analysis of online data related to food by means of social network analysis, data mining, and sentiment analysis. Media content, antecedents, and contingencies regarding the analysis of online food related data are analyzed.

**Table 9:** Top ten factor 7 keywords and publications addressing the analysis of online food related data

Factor 7. Variance explained: 2.14%		
Keyword	# of mentions	FL
Sentiment_analysis	35	0.74
Tweet	14	0.713
Halal	11	0.704
Datum	35	0.694
Detection	12	0.686
Twitter	102	0.684
Topic	19	0.679
Mining	20	0.641
Classification	12	0.612
Sentiment	15	0.61
Other 14 Keywords* with FL ≥ 0.591		

Representative publications	# referenced keywords
Mostafa (2021) J. Int. Consum. Mark	10
Mostafa (2019) Int. J. Market Res	7
Rimjhim et al. (2020) IEEE Trans. Comput. Soc. Syst	7
Zhang et al. (2020) ISPRS Int. Geo-Inf	7
Ullah et al. (2021) Expert Syst. Appl	6
Mostafa (2022) Food Cult. Soc	6
Koylu (2019) Int. J. Geogr. Inf. Sci	6

*Further keywords (sorted by decreasing FL): analytic; text; modelling; data_mining; muslim; geography; analysis; spatial; surveillance; machine; social_network; learning; world; disaster

Many studies in this research stream emphasize the use of different methods and tools to analyze online communication data. By using opinion mining techniques, Mostafa (2019) analyzed food sentiments regarding halal food expressed on Twitter detecting a generally positive sentiment toward halal food, as well as a heterogeneous group of halal food consumers divisible by concern for food authenticity, self-identity, animal welfare attitudes, and level of religiosity. By using social network analysis Mostafa (2021) examined the structure, dynamics, and influencers in halal food networks, founding that few social mediators or “influencers” control the diffusion of information through a small world preferential attachment network that links digital halal food consumers. The same author analyzed Wikipedia’s clickstream data in order to study users’ halal food navigation strategies on Wikipedia servers discovering that only a few articles or “influencers” within close-knot communities control the flow of halal food information (Mostafa 2022).

As well the use of geocoding has an important place in this research stream. By using geocoding, Rimjhim et al. (2020) analyzed data from Twitter and Wikipedia, to know how the conversational discourse on online social networks vary semantically and geographically over time finding that although there is a significant homogenization in online discussion topics, despite geographical distance, it is not similar across all topics of discussion and location. Zhang et al. (2020) explored individuals’ emotions and cognition of cultural food differences among people from South and North China by using the machine learning method of natural language processing (NLP) by posting on the Zhihu Q&A platform the question “What are the differences between South and North China that you ever know?” They found that food culture is the most popular difference among people from North and South China and that individuals tend to have a negative attitude toward food cultures that differ from their own. Analyzing geo-located and reciprocal user mention and reply tweets over the course of the 2016 primary and presidential elections in the United States, Koylu (2019) found that the discourse was divided between election-related discussions of the political campaigns and candidates, and civil rights, being the last the more dominant. Ullah et al. (2021) propose an architecture to store data to accelerate the development process of the machine learning classifiers using rule-based and logistic regression.

The contribution of this research stream to the social sciences lies, without doubt, in the novel approaches to analyzing online data. Further research could extend the use of these tools in their research or propose new ones. And, since most studies analyze text, it is recommended the development of tools to analyze images.

#### Research stream on “online communication and eating disorders” (Factor 9)

The ninth research stream explains 1.76% of the variance of keyword relationships. *Blog* and *site* were the most often listed keywords (62 and 38 mentions), but in terms of factor loadings, the stream was best represented by the keywords *discourse* (FL = 0.557)*,* followed by *blog.* An inspection of the remaining seventeen keywords, confirms the eating disorders approach. Hence, this research stream studies the topic of “online communication and eating disorders.”

Without a leading theory, representative publications of this research stream (see Table 10) analyze online communication related to eating disorders, sharing the same discourse. Articles address online communication related to eating disorders by means of virtual ethnography, netnography, and interpretative phenomenological analysis, among others. They analyze web and social media content as well as antecedents and contingencies regarding online communication and eating disorders.

**Table 10:** Top ten factor 9 keywords and publications addressing online communication and eating disorders

Factor 9. Variance explained: 1.76%		
Keyword	# of mentions	FL
Discourse	14	0.557
Blog	62	0.522
Body-image	14	0.486
Eating_disorder	26	0.476
Body	20	0.466
Politics	35	0.466
Narrative	11	0.463
Anorexia	9	0.445
Site	38	0.393
Nervosa	9	0.377
Other 8 Keywords* with FL ≥ 0.375		

Representative publications	# referenced keywords
Cinquegrani and Brown (2018) Qual. Res. Sport Exerc. Health	10
Lavis (2017) Geoforum	5
Herrick et al. (2021) Int. J. Eating Disord	5
Yeo (2014) Environ. Commun	4
Costa et al. (2019) Appetite	4
Sikka (2019) Food Cult. Soc	4

*Further keywords (sorted by decreasing FL): ethnography; culture; participation; platform; identity; ethic; street; self

Some studies in this research stream explore online narratives, experiences, and discussions regarding eating disorders (ED) online. By using content analysis of ‘food porn’ websites and blogs, as well as participant observation and interviews regarding ‘pro-anorexia’ websites, Lavis (2017) found that participants “eat” in, and through cyberspace, beyond and among bodies. Cinquegrani and Brown (2018) explored narratives of experiences and conceptualizations through online social media forums regarding the eating disorder Orthorexia Nervosa (ON), a fixation on eating proper food accompanied by excessive exercise. The authors found three main narratives: pursuit (i.e. the individuals are on a quest to ‘better’ themselves), resistance to the illness narrative, and the recovery (i.e. after accepting the ‘illness narrative’). The authors suggest considering ON a lifestyle syndrome embodied in social and cultural processes. By analyzing TikTok posts containing the hashtag (#) EDrecovery, Herrick et al. (2021) concluded that creators share their personal experiences with recovery by using popular (or viral) video formats, succinct storytelling, and the production of educational content.

Other studies explored online conversations in order to understand how individuals confer value and meaning to ‘healthy’ eating behaviors. Consumers are active co-producers of value and meaning regarding the impact of green products on their health and the environment, and their understanding of health and sustainability is affected by cultural meanings and pleasure, which lead them to attribute additional unsubstantiated traits to certain products ascribed as virtuous (Yeo 2014). Examining the visual and textual framings of ‘superfoods’ on social media, it was found that superfoods are a marker of idealized identity mobilized by using postfeminist, neoliberal, and food justice discourses (Sikka 2019), the healing potential of veganism is derived from a passionate investment of the self that redefines young women’s ways of being in the world (Costa et al. 2019).

In sum, this research contributes to the understanding of the complexity of eating disorders by uncovering the processes and meanings of eating disorders and how they are portraited online. Some studies in this research stream also discloses how individuals confer meaning to healthy eating behaviors and how an idealized identity ascribes virtuous attributes to some foods. Further research could explore if this initially idealized identity of healthy foods leads to future eating disorders.

### Service industry discourse on “food online reviews in the service industry” (Factor 2)

One research stream was found in this cluster, which possesses an integrative discourse: “food online reviews in the service industry.” This research stream explains 9.87% of the variance of keyword relationships, indicating a research stream of second-highest distinction. While *word-of-mouth* and *satisfaction* were the most often listed keywords (77 and 60 mentions), the research stream was best represented (in terms of factor loadings) by the keywords *hotel* (FL = 0.868)*,* followed by (*online) reviews. Performance* and *(consumer) satisfaction* related to *restaurant, service, hospitality* constitute the remainders of the top ten keywords. An inspection of the remaining 49 keywords confirms this focus on application-oriented topics from the perspective of the service industry. Thus, this research stream addresses the topic “food online reviews in the service industry.”

Representative publications of this research stream (see Table 11) reference each more than 10 keywords of factor 2. Regarding theories and conceptualizations, most of the articles refer to electronic word of mouth (e-WOM) and online review. Thus, an inclusive and shared research discourse can be diagnosed.

**Table 11:** Top ten factor 2 keywords and publications addressing food online reviews in the service industry

Factor 2. Variance explained: 9.87%		
Keyword	# of mentions	FL
Hotel	21	0.868
Online_reviews	25	0.868
word-of-mouth	77	0.862
Review	46	0.845
Performance	32	0.836
Satisfaction	60	0.831
Restaurant	44	0.814
Service	47	0.808
Hospitality	34	0.800
Customer_satisfaction	21	0.799
Other 49 Keywords* with FL ≥ 0.793		

Representative publications	# referenced keywords
Abrudan et al. (2020) Sustainability	16
Kim et al. (2016) Int. J. Hosp. Manag	15
Liu et al. (2020) Int. J. Contemp. Hosp. Manag	14
Cambra-Fierro et al. (2020) Corp. Soc. Responsib. Environ. Manag	13
Bilgihan et al. (2018) J. Hosp. Market. Manag	13
Zinko et al. (2021) J. Theor. Appl. Electron. Commer. Res	13
Phillips et al. (2017) J. Travel Res	13

*Further keywords (sorted by decreasing FL): customer; experience; quality; tourism; ewom; loyalty; user-generated_content; attribute; tripadvisor; impact; antecedent; electronic; perceive; brand; sale; value; product; motivation; content; word; online; big_data; model; moderate; management; role; mouth; industry; sector; corporate; engagement; decision; involvement; shopping; orientation; recovery; user; generation; co-creation; critical; strategy; development; relationship; new; social-responsibility; corporate_social_responsibility; facebook; negative; visual

A closer look at these articles (selected by maximum number of referenced keywords) provides insights about the methods applied and types of insights gained from this research discourse (Table 11 right columns). These articles address online food reviews as an indicator of service quality, linking both by means of regression analysis or structural equation modeling. Antecedents, consequences as well as contingencies of online food reviews are analyzed.

In a narrow effects perspective, Kim et al. (2016) found that the number of online reviews correlates with restaurant performance. By analyzing online customer comments on Yelp.com, Bilgihan et al. (2018) found that a focus on selected menu offerings, food, ambiance, and service can create buzz in social media. Addressing the broader scope of tourism industry, Abrudan et al. (2020) studied customer review scores on booking.com to analyze the impact of different hotel facilities on customers’ overall ratings, confirming the special relevance of food service for hotel ratings. Another analysis of online reviews from 68 online platforms however did not confirm such a special relevance of food services, with hotel attributes, including quality of rooms, Internet provision, and building to impact hotel performance most (Phillips et al. 2017). Altogether, these works highlight the importance of food reviews as drivers of positive consumer feedback primarily in the restaurant industry but less so in the broader hospitality industry.

Other works critically reflect on the antecedents of consumers’ online food reviews. Investigating consumers´ personal drivers to write food reviews, Liu et al. (2020) found that personal motivation, and especially altruism, influences the posting of negative consumer online reviews. Cambra-Fierro et al. (2020) discovered that a company’s corporate social responsibility can steer consumers to identify and link themselves to brands generating buy-back and recommendation behaviors. These works thus reveal behavioral drivers on the creation of food reviews both at the consumer and company level. Finally, several works investigate contingencies regarding the effects of food reviews: Zinko et al. (2021) found that reviewer-submitted (food) images influence consumers’ attitudes only when they are consistent with the review text. This contingency perspective on the effects of food reviews in social media seems the more needed given that previous research, as outlined above, came to divergent conclusions about the impact of online food reviews on consumers’ service ratings.

With most articles in this research stream addressing written food reviews online on different social media, further research might analyze not just the use of written messages, but as well the use of images in online reviews.

### Patterns of the overall research system

**Fig. 2:**
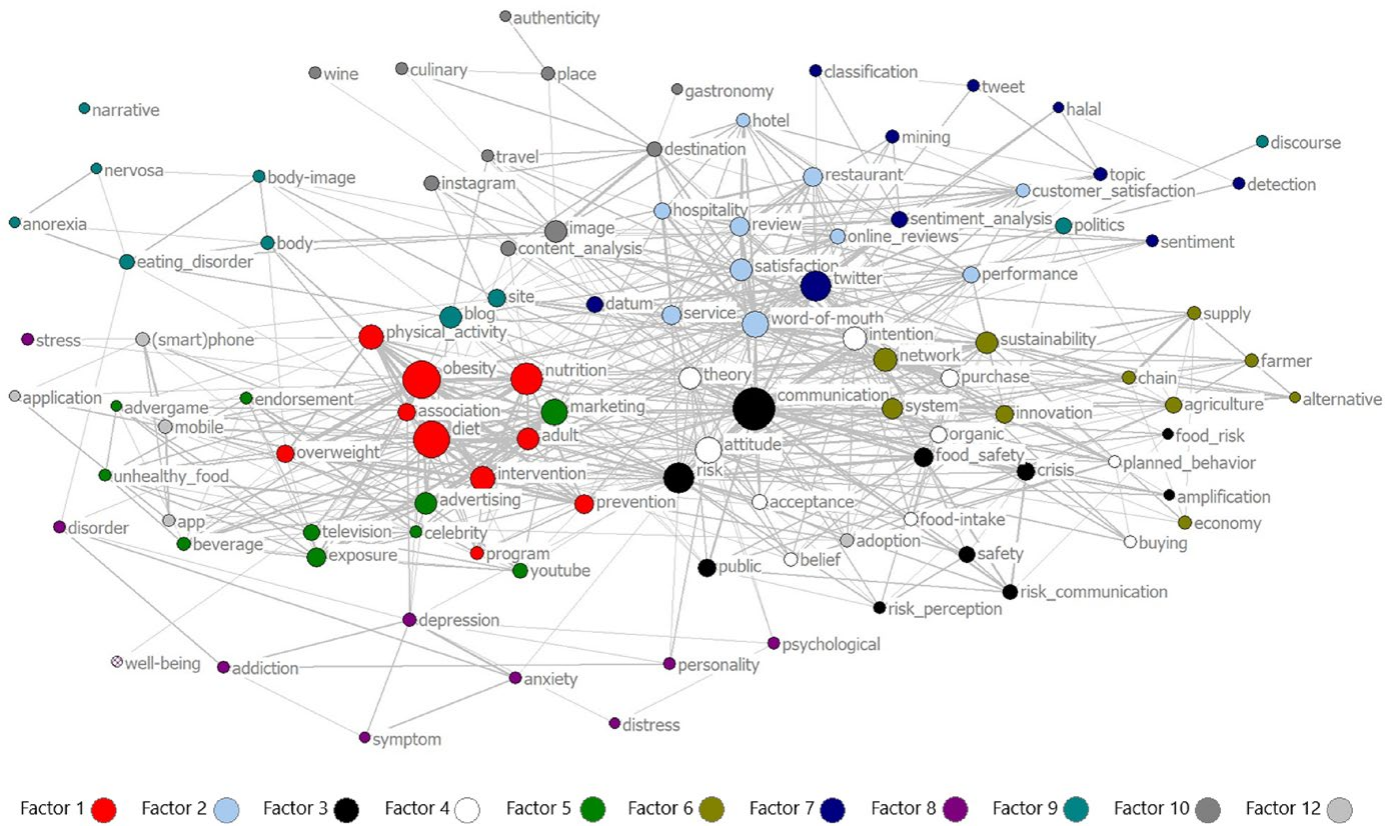
Network Visualization of Factors´ Top-10-Keywords Relations

The previous analyses were restricted to the level of single research streams. To complement this perspective, the relationship between research streams is analyzed by means of a network analysis. Hereto, a multidimensional scaling of the linkages of the top-ten keywords per factor is calculated and visualized in Fig. 2. While the size of nodes displays the relative mentioning frequency of each keyword, their positioning within the figure informs about their overall centrality and connectedness. Although the largest nodes or most often mentioned keywords are *communication, diet, risk,* and *obesity*, this chart indicates a clear focality on the keyword *communication*.

The closeness of single keywords indicates their relationship with each other, and with other research streams. To ease interpretation, each factors’ keywords are marked in different colors. Thus, the distance between keywords stemming from different research streams reveals not only their closeness but as well interconnections between their respective research streams. For example, *obesity* and *diet* are closely linked to *advertising*. This implies close connections between the discourses on “Online Tools for Healthy Diet Intervention Programs” (factor 1, marked in red) and “Online Food Advertising Exposure” (factor 5, marked in dark green). While these two discourses assume a different actor perspective, zooming into consumers’ or marketers’ interest, they nonetheless discuss related topics from a complementary perspective.

In contrast, a large distance among words or factors shows a weak relationship or missing links between research streams; for example, a large distance can be observed among keywords related to “Sustainable Food Communication Online” (factor 6) and to “Social Media and Food Tourism” (factor 10). This shows that these two research streams are not yet strongly related. Future research might contribute by linking those different perspectives together.

Furthermore, the location of keywords related to “Social Media and Mental Disorders” (factor 8) at the outer skirt of the figure reveals that this research stream is a truly peripheral discourse. Finally, the method-driven discourse on “Food Online Reviews in the Service Industry” (factor 2) is clearly more related to the core discourse, to *twitter* and the different methods of analysis.

## Conclusions and implications

This study presents a bibliometric analysis of the research conducted regarding food and social media within the social sciences. By using co-word analysis, this study evaluated 413 main Keywords contained in 1356 articles by means of factor and social network analysis. The study shows that the number of studies conducted on this topic has increased rapidly, indicating a growing interest in food and social media. Besides, the results reveal four main research clusters (i.e. Psychological Research Realm, Action-Oriented Research, Broader Communication Issues, and Service Industry Discourse) containing the main topics of research.

The Psychological Research Cluster analyzes online tools for healthy diet intervention programs, the use of apps for service purposes or dietary change, the exposure of children and adolescents to influencer marketing in YouTube videos, as well as the antecedents and consequences of social media use and mental disorders. The Action-Oriented Research cluster analyzes online food risk communication, behavioral intention and buying online, as well as the use of social media for food tourism. The Broader Communication Issues cluster studies sustainable food communication online, online food related data, and the relationship between online communication and eating disorders. Finally, the Service Industry Discourse cluster explores online reviews in the service industry.

Future research could transfer topics in order to have a broad scope of research. For example, the insights gained on the discourse “food and the use of apps” (factor 12), could be transferred to studies regarding “online food risk communication” (factor 3). A further alternative is to transfer the potential of the sophisticated text-mining as method of analysis used in the discourse “analysis of online food related data” (factor 7) enriched by picture mining, in order to address research questions related to how food is perceived and marketed (e.g. factor 6). Another possibility is to intersect, for example, the topic of factor 1, which addresses more positive psychological constructs in detail, and factor 8, which addresses topics more related to clinical psychology. Further integration of theoretical models stemming from psychology (e.g. factor 1 and factor 2) into the practically oriented joint discourse on service industry setting (Factor 2). More theoretical foundations might help to generate broader insights. Other studies could compare target groups (e.g. comparing adults, adolescents, and children), in different countries, regarding the same topics (e.g. fast-food intake while consuming social media). Additionally, the analysis of texts or reviews could be enriched through the analysis images, or by developing tools to analyze images. Other ideas are summarized in Table 12, and elaborated in the discussion of the single research streams above.

**Table 12:** Main Clusters, research streams' characteristics, and recommendations for future research

Cluster and Research Streams	Main Focus	Possible Future Research Directions	
Psychological Research Realm	Online Tools for Healthy Diet Intervention Programs (Factor 1)	Use of online tools for healthy diet intervention programs, including protocols of novel internet-based intervention tools, and the evaluation of established online tools	Use of social media or development of novel tools, to target parents and children separately, delivering content designed especially for each group
	Online Food Advertising Exposure (Factor 5)	Children and adolescents’ exposure to influencer marketing in YouTube videos, mainly	Promotion of healthy foods by influencers on different social media with video content (e.g. TikTok, Instagram), comparing different target groups (e.g. adults, adolescents, and children)
	Social Media and Mental Disorders (Factor 8)	Antecedents and consequences of social media use and mental disorders	Use of social media for mental health
	Food and the Use of Apps (Factor 12)	Use of smartphone apps for service purposes, or for dietary change	Comparative studies among apps, with different target groups in different countries
Action-Oriented Research	Online Food Risk Communication (Factor 3)	Coverage of topics related to health risk, antecedents of consumers´ food risk information seeking, and the impact of online sources on consumers´ risk perception	How social media could be used effectively for public health and food safety risk by using quantitative and qualitative methods of research
	Behavioral Intention and Buying Online (Factor 4)	Influence of social media on behavioral intention and buying online green products	Use of mixed methods to reduce the intention-behavior gap
	Social Media and Food Tourism (Factor 10)	Analysis of social media content as well as antecedents and contingencies regarding social media and food tourism for destination image and its promotion	Analysis of further social media tools in addition to Instagram, Tripadvisor, and TikTok. As well as comparisons among tools and countries
Broader Communication Issues	Sustainable Food Communication Online (Factor 6)	Sustainable food communications online among participants of Alternative Food Networks, mainly	How social media communications influence behavior-change and sustainable food consumption among consumers
	Analysis of Online Food Related Data (Factor 7)	Analysis of online food related data	Further research could extend the use of these tools or propose new ones. Developing tools to analyze images
	Online Communication and Eating Disorders (Factor 9)	Web and social media content as well as antecedents and contingencies regarding online communication and eating disorders	Further research could explore if this initially idealized identity of healthy foods, leads to future eating disorders
Service Industry Discourse	Food Online Reviews in the Service Industry (Factor 2)	Online food reviews as an indicator of service quality, drivers of positive and negative consumer feedback, their correlation with restaurant performance and their effects on social media	Analysis of images in online reviews

By suggesting future research directions, this study help scholars to find relevant future topics of research in this area of study. The findings presented in this study can be beneficial for marketing and business scholars, as well as companies, and organizations interested in understanding the relationships between food and social media.

## Supplementary Information

Below is the link to the electronic supplementary material.

Supplementary file1 (PDF 614 kb)

## Data Availability

On request.
