# Supplementary material for: Food and social media: a research stream analysis
Source: Manag Rev Q. 2023 Feb 18:1–39. Online ahead of print. doi: 10.1007/s11301-023-00330-y (PMC9938734; doi:10.1007/s11301-023-00330-y)
Supplement: Supplementary file 1 — Supplementary file1 (PDF 614 kb) [file 11301_2023_330_MOESM1_ESM.pdf]

Supplementary Material

Table S1: Force pick up words.

social media marketing	fast food	public health
natural language processing	unhealthy food	qualitative research
corporate social responsibility	food risk	randomized controlled-trial
user-generated content	food safety	risk communication
behavior change	food security	risk perception
big data	junk food	sentiment analysis
consumer behavior	nutrition education	social media
content analysis	online reviews	social network
customer satisfaction	physical activity	social marketing
data mining	planned behavior	young adult
eating disorder		

Table S2: Code words.

*communication	*waste	*consumer_behavior
communication	waste	consumer_behavior | consumer-behavior
*consumption	*program	*evaluation
consumption	program	evaluation
*obesity	*medical	*food_security
obesity	medical | medicine | pharmaceutical	food_security
*child	*message	*assessment
child | childhood	message	assessment
*online	*response	*halal
online	response	halal
*impact	*behavioral	*emerge
impact	behavioral | behavioural	emerge
*diet	*perspective	*awareness
diet | dietary | dietary-intake	perspective	awareness
*media	*vegetable	*factor
media	vegetable	factor
*tourism	*(smart)phone	*family
tourism | tourist	phone | smartphone	family
*nutrition	*design	*classification
nutrition	design	classification
*social	*green	*status
social	green	status
*model	*adoption	*personal
model	adoption	personal
*adolescent	*economy	*population
adolescence | adolescent	economy	population
*twitter	*scale	*sale
twitter	scale	sale
*internet	*machine	*self-efficacy
internet	machine	self-efficacy
*risk	*user-generated_content	*trial
risk	user-generated_content	trial
*perception	*climate	*video
perception	climate | climate-change	video
*management	*hotel	*outcome
management	hotel	outcome
*product	*group	*weight-loss
product	group	weight-loss
*quality	*belief	*power
quality	belief	power
*marketing	*metaanalysis	*loss
marketing	metaanalysis	loss
*eating	*topic	*availability
eat | eating	topic	availability
*environment	*united-state	*critical
environment | environmental	united-state	critical
*facebook	*space	*culinary
facebook	space	culinary
*word-of-mouth	*body	*identification
word-of-mouth	body	identification
*social_network	*barrier	*habit
social_network	barrier	habit
*experience	*influencer	*nervosa
experience | experiential	influencer	nervosa
*community	*beverage	*randomized_controlled-trial
community	beverage	randomized_controlled-trial
*advertising	*illness	*virtual
advertisement | advertising	illness	virtual
*intervention	*ewom	*tool
intervention	ewom	tool

<table>
<tr><td>*physical_activity</td><td>*time</td><td>*taste</td></tr>
<tr><td>physical_activity | physical-activity</td><td>time</td><td>taste</td></tr>
<tr><td>*attitude</td><td>*supply</td><td>*social_marketing</td></tr>
<tr><td>attitude</td><td>supply</td><td>social_marketing</td></tr>
<tr><td>*smoking</td><td>*people</td><td>*photography</td></tr>
<tr><td>cigarette | e-cigarette | smoking | tobacco</td><td>people</td><td>photography</td></tr>
<tr><td>*intention</td><td>*sharing</td><td>*meal</td></tr>
<tr><td>intention</td><td>share | sharing</td><td>meal</td></tr>
<tr><td>*network</td><td>*behavior_change</td><td>*mass</td></tr>
<tr><td>network</td><td>behavior_change | behavior-change</td><td>mass</td></tr>
<tr><td>*knowledge</td><td>*farmer</td><td>*adverse</td></tr>
<tr><td>knowledge</td><td>farmer</td><td>adverse</td></tr>
<tr><td>*use</td><td>*energy</td><td>*gratification</td></tr>
<tr><td>usage | use</td><td>energy</td><td>gratification</td></tr>
<tr><td>*analysis</td><td>*drinking</td><td>*change</td></tr>
<tr><td>analysis</td><td>drink | drinking</td><td>change</td></tr>
<tr><td>*brand</td><td>*corporate</td><td>*governance</td></tr>
<tr><td>brand</td><td>corporate</td><td>governance</td></tr>
<tr><td>*technology</td><td>*allergy</td><td>*home</td></tr>
<tr><td>technology</td><td>allergy</td><td>home</td></tr>
<tr><td>*sustainability</td><td>*role</td><td>*adherence</td></tr>
<tr><td>sustainability | sustainable</td><td>role</td><td>adherence</td></tr>
<tr><td>*image</td><td>*opinion</td><td>*country</td></tr>
<tr><td>image</td><td>opinion</td><td>country</td></tr>
<tr><td>*value</td><td>*meat</td><td>*body-mass</td></tr>
<tr><td>value</td><td>meat</td><td>body-mass</td></tr>
<tr><td>*satisfaction</td><td>*modify</td><td>*genetically</td></tr>
<tr><td>satisfaction</td><td>modify</td><td>genetically</td></tr>
<tr><td>*gender</td><td>*qualitative_research</td><td>*detection</td></tr>
<tr><td>gender | girl | woman</td><td>qualitative_research</td><td>detection</td></tr>
<tr><td>*theory</td><td>*customer_satisfaction</td><td>*mouth</td></tr>
<tr><td>theory</td><td>customer_satisfaction</td><td>mouth</td></tr>
<tr><td>*culture</td><td>*index</td><td>*shopping</td></tr>
<tr><td>cultural | culture</td><td>index</td><td>shopping</td></tr>
<tr><td>*trust</td><td>*business</td><td>*narrative</td></tr>
<tr><td>trust</td><td>business</td><td>narrative</td></tr>
<tr><td>*blog</td><td>*benefit</td><td>*rural</td></tr>
<tr><td>blog | blogg</td><td>benefit</td><td>rural</td></tr>
<tr><td>*adult</td><td>*event</td><td>*pregnancy</td></tr>
<tr><td>adult | adulthood</td><td>event</td><td>pregnancy</td></tr>
<tr><td>*young_adult</td><td>*framework</td><td>*risk-factor</td></tr>
<tr><td>young_adult | young-adult | youth</td><td>framework</td><td>risk-factor</td></tr>
<tr><td>*system</td><td>*loyalty</td><td>*fear</td></tr>
<tr><td>system</td><td>loyalty</td><td>fear</td></tr>
<tr><td>*healthy</td><td>*low-income</td><td>*generation</td></tr>
<tr><td>healthy</td><td>low-income</td><td>generation</td></tr>
<tr><td>*engagement</td><td>*participation</td><td>*application</td></tr>
<tr><td>engagement</td><td>participation</td><td>application</td></tr>
<tr><td>*service</td><td>*young</td><td>*commerce</td></tr>
<tr><td>service</td><td>young</td><td>commerce</td></tr>
<tr><td>*covid-19</td><td>*mining</td><td>*advergame</td></tr>
<tr><td>coronavirus | covid-19 | pandemic</td><td>mining</td><td>advergame</td></tr>
<tr><td>*digital</td><td>*seek</td><td>*alternative</td></tr>
<tr><td>digital</td><td>seek</td><td>alternative</td></tr>
<tr><td>*review</td><td>*care</td><td>*coverage</td></tr>
<tr><td>review</td><td>care</td><td>coverage</td></tr>
<tr><td>*perceive</td><td>*influence</td><td>*equation</td></tr>
<tr><td>perceive</td><td>influence</td><td>equation</td></tr>
<tr><td>*food_safety</td><td>*control</td><td>*gastronomy</td></tr>
<tr><td>food_safety</td><td>control | controlled</td><td>gastronomy</td></tr>
<tr><td>*practice</td><td>*place</td><td>*ethnography</td></tr>
<tr><td>practice</td><td>place</td><td>ethnography</td></tr>
<tr><td>*choice</td><td>*user</td><td>*activism</td></tr>
<tr><td>choice</td><td>user</td><td>activism</td></tr>
<tr><td>*web</td><td>*moderate</td><td>*cue</td></tr>
<tr><td>web | website</td><td>moderate</td><td>cue</td></tr>
<tr><td>*disease</td><td>*depression</td><td>*world</td></tr>
<tr><td>disease</td><td>depression</td><td>world</td></tr>
<tr><td>*overweight</td><td>*cooking</td><td>*willingness</td></tr>
<tr><td>overweight</td><td>cooking</td><td>willingness</td></tr>
<tr><td>*prevention</td><td>*anxiety</td><td>*poverty</td></tr>
<tr><td>prevention</td><td>anxiety</td><td>poverty</td></tr>
<tr><td>*crisis</td><td>*content</td><td>*stigma</td></tr>
<tr><td>crisis</td><td>content</td><td>stigma</td></tr>
<tr><td>*innovation</td><td>*intake</td><td>*organization</td></tr>
<tr><td>innovation</td><td>intake</td><td>organization</td></tr>
<tr><td>*survey</td><td>*discourse</td><td>*tripadvisor</td></tr>
<tr><td>questionnaire | survey</td><td>discourse</td><td>tripadvisor</td></tr>
<tr><td>*strategy</td><td>*data_mining</td><td>*validity</td></tr>
<tr><td>strategy</td><td>data_mining | natural_language_processing</td><td>validity</td></tr>
</table>

<table>
<tr><td>*policy</td><td>*word</td><td>*university</td></tr>
<tr><td>policy</td><td>word</td><td>university</td></tr>
<tr><td>*restaurant</td><td>*travel</td><td>*state</td></tr>
<tr><td>restaurant</td><td>travel</td><td>state</td></tr>
<tr><td>*education</td><td>*norm</td><td>*muslim</td></tr>
<tr><td>education</td><td>norm</td><td>muslim</td></tr>
<tr><td>*exposure</td><td>*falsehood</td><td>*monitoring</td></tr>
<tr><td>exposure</td><td>misinformation | rumor</td><td>monitoring</td></tr>
<tr><td>*science</td><td>*planned_behavior</td><td>*visual</td></tr>
<tr><td>science</td><td>planned_behavior</td><td>visual</td></tr>
<tr><td>*public</td><td>*peer</td><td>*reduction</td></tr>
<tr><td>public</td><td>peer</td><td>reduction</td></tr>
<tr><td>*support</td><td>*structural</td><td>*prescription</td></tr>
<tr><td>support</td><td>structural</td><td>prescription</td></tr>
<tr><td>*association</td><td>*co-creation</td><td>*social-responsibility</td></tr>
<tr><td>association</td><td>co-creation</td><td>social-responsibility</td></tr>
<tr><td>*weight</td><td>*ethic</td><td>*need</td></tr>
<tr><td>weight</td><td>ethic | moral</td><td>need</td></tr>
<tr><td>*organic</td><td>*food-intake</td><td>*orientation</td></tr>
<tr><td>organic</td><td>food-consumption | food-intake</td><td>orientation</td></tr>
<tr><td>*sentiment_analysis</td><td>*disorder</td><td>*negative</td></tr>
<tr><td>sentiment_analysis</td><td>disorder</td><td>negative</td></tr>
<tr><td>*politics</td><td>*celebrity</td><td>*spatial</td></tr>
<tr><td>political | politics</td><td>celebrity</td><td>spatial</td></tr>
<tr><td>*emotion</td><td>*validation</td><td>*relationship</td></tr>
<tr><td>emotion | emotional</td><td>validation</td><td>relationship</td></tr>
<tr><td>*literacy</td><td>*personality</td><td>*ehealth</td></tr>
<tr><td>literacy</td><td>personality</td><td>ehealth</td></tr>
<tr><td>*purchase</td><td>*risk_perception</td><td>*inequality</td></tr>
<tr><td>purchase</td><td>risk_perception</td><td>inequality</td></tr>
<tr><td>*promotion</td><td>*school</td><td>*decision-making</td></tr>
<tr><td>promotion</td><td>school</td><td>decision-making</td></tr>
<tr><td>*site</td><td>*global</td><td>*action</td></tr>
<tr><td>site</td><td>global | globalization</td><td>action</td></tr>
<tr><td>*prevalence</td><td>*addiction</td><td>*authenticity</td></tr>
<tr><td>prevalence</td><td>addiction</td><td>authenticity</td></tr>
<tr><td>*agriculture</td><td>*frame</td><td>*concern</td></tr>
<tr><td>agricultural | agriculture</td><td>frame</td><td>concern</td></tr>
<tr><td>*public_health</td><td>*buying</td><td>*build</td></tr>
<tr><td>public_health | public-health</td><td>buy | buying</td><td>build</td></tr>
<tr><td>*research</td><td>*endorsement</td><td>*amplification</td></tr>
<tr><td>research</td><td>endorsement</td><td>amplification</td></tr>
<tr><td>*datum</td><td>*infant</td><td>*individual</td></tr>
<tr><td>datum</td><td>infant</td><td>individual</td></tr>
<tr><td>*news</td><td>*issue</td><td>*fda</td></tr>
<tr><td>news</td><td>issue</td><td>fda</td></tr>
<tr><td>*performance</td><td>*nutrition_education</td><td>*australium</td></tr>
<tr><td>performance</td><td>nutrition_education</td><td>australium</td></tr>
<tr><td>*preference</td><td>*patient</td><td>*effect</td></tr>
<tr><td>preference</td><td>patient</td><td>effect</td></tr>
<tr><td>*lifestyle</td><td>*self</td><td>*anorexia</td></tr>
<tr><td>lifestyle | life-style</td><td>self</td><td>anorexia</td></tr>
<tr><td>*market</td><td>*source</td><td>*cancer</td></tr>
<tr><td>market</td><td>source</td><td>cancer</td></tr>
<tr><td>*destination</td><td>*mother</td><td>*neighborhood</td></tr>
<tr><td>destination</td><td>mother</td><td>neighborhood</td></tr>
<tr><td>*television</td><td>*trend</td><td>*unite</td></tr>
<tr><td>television</td><td>trend</td><td>unite</td></tr>
<tr><td>*feeding</td><td>*city</td><td>*netnography</td></tr>
<tr><td>feed | feeding</td><td>city</td><td>netnography</td></tr>
<tr><td>*determinant</td><td>*exercise</td><td>*social_media_marketing</td></tr>
<tr><td>determinant</td><td>exercise</td><td>social_media_marketing</td></tr>
<tr><td>*hospitality</td><td>*disaster</td><td>*popularity</td></tr>
<tr><td>hospitality</td><td>disaster</td><td>popularity</td></tr>
<tr><td>*development</td><td>*wine</td><td>*south</td></tr>
<tr><td>development</td><td>wine</td><td>south</td></tr>
<tr><td>*identity</td><td>*vegan_vegetarian</td><td>*well-being</td></tr>
<tr><td>identity</td><td>vegan | vegetarian</td><td>well-being</td></tr>
<tr><td>*study</td><td>*stress</td><td>*sport</td></tr>
<tr><td>study</td><td>stress</td><td>sport</td></tr>
<tr><td>*safety</td><td>*resource</td><td>*process</td></tr>
<tr><td>safety</td><td>resource</td><td>process</td></tr>
<tr><td>*qualitative</td><td>*self-management</td><td>*street</td></tr>
<tr><td>qualitative</td><td>self-management | self-regulation</td><td>street</td></tr>
<tr><td>*industry</td><td>*mental</td><td>*natural</td></tr>
<tr><td>industry</td><td>mental</td><td>natural</td></tr>
<tr><td>*drug</td><td>*sentiment</td><td>*recipe</td></tr>
<tr><td>drug</td><td>sentiment</td><td>recipe</td></tr>
<tr><td>*motivation</td><td>*store</td><td>*sector</td></tr>
<tr><td>motivation</td><td>store | supermarket</td><td>sector</td></tr>
</table>

<table>
<tr><td>*learning</td><td>*new</td><td>*recovery</td></tr>
<tr><td>learning</td><td>new</td><td>recovery</td></tr>
<tr><td>*acceptance</td><td>*unhealthy_food</td><td>*warning</td></tr>
<tr><td>acceptance</td><td>unhealthy_food</td><td>warning</td></tr>
<tr><td>*instagram</td><td>*traditional</td><td>*symptom</td></tr>
<tr><td>instagram</td><td>traditional</td><td>symptom</td></tr>
<tr><td>*parent</td><td>*involvement</td><td>*sensitivity</td></tr>
<tr><td>parent | parental | parenting</td><td>involvement</td><td>sensitivity</td></tr>
<tr><td>*pattern</td><td>*antecedent</td><td>*resistance</td></tr>
<tr><td>pattern</td><td>antecedent</td><td>resistance</td></tr>
<tr><td>*local</td><td>*age</td><td>*work</td></tr>
<tr><td>local</td><td>age</td><td>work</td></tr>
<tr><td>*big_data</td><td>*bias</td><td>*trade</td></tr>
<tr><td>big_data</td><td>bias</td><td>trade</td></tr>
<tr><td>*content_analysis</td><td>*decision</td><td>*platform</td></tr>
<tr><td>content_analysis</td><td>decision</td><td>platform</td></tr>
<tr><td>*fast-food</td><td>*life</td><td>*reputation</td></tr>
<tr><td>fast_food | fast-food | junk_food</td><td>life</td><td>reputation</td></tr>
<tr><td>*eating_disorder</td><td>*ethnic</td><td>*acculturation</td></tr>
<tr><td>eating_disorder | eating-disorder</td><td>ethnic | race</td><td>acculturation</td></tr>
<tr><td>*youtube</td><td>*corporate_social_responsibility</td><td>*citizen</td></tr>
<tr><td>youtube</td><td>corporate_social_responsibility | csr</td><td>citizen</td></tr>
<tr><td>*fruit</td><td>*focus</td><td>*infection</td></tr>
<tr><td>fruit</td><td>focus</td><td>infection</td></tr>
<tr><td>*method</td><td>*app</td><td>*entrepreneurship</td></tr>
<tr><td>method | methodology</td><td>app</td><td>entrepreneurship</td></tr>
<tr><td>*modeling</td><td>*foodborne</td><td>*justice</td></tr>
<tr><td>modeling | modelling</td><td>foodborne</td><td>justice</td></tr>
<tr><td>*student</td><td>*weight-gain</td><td>*fit</td></tr>
<tr><td>student</td><td>weight-gain</td><td>fit</td></tr>
<tr><td>*online_reviews</td><td>*psychological</td><td>*attribute</td></tr>
<tr><td>online_reviews</td><td>psychological</td><td>attribute</td></tr>
<tr><td>*customer</td><td>*nutritional</td><td>*food_risk</td></tr>
<tr><td>customer</td><td>nutritional</td><td>food_risk</td></tr>
<tr><td>*credibility</td><td>*sugar</td><td>*cognitive</td></tr>
<tr><td>credibility</td><td>sugar | sugar-sweetened</td><td>cognitive</td></tr>
<tr><td>*chain</td><td>*tweet</td><td>*efficacy</td></tr>
<tr><td>chain</td><td>tweet</td><td>efficacy</td></tr>
<tr><td>*analytic</td><td>*surveillance</td><td>*disparity</td></tr>
<tr><td>analytic</td><td>surveillance</td><td>disparity</td></tr>
<tr><td>*china</td><td>*movement</td><td>*dynamics</td></tr>
<tr><td>china</td><td>movement</td><td>dynamics</td></tr>
<tr><td>*label</td><td>*recommendation</td><td>*distress</td></tr>
<tr><td>label</td><td>recommendation</td><td>distress</td></tr>
<tr><td>*electronic</td><td>*geography</td><td>*campaign</td></tr>
<tr><td>electronic</td><td>geography</td><td>campaign</td></tr>
<tr><td>*urban</td><td>*interaction</td><td>*attention</td></tr>
<tr><td>urban</td><td>interaction</td><td>attention</td></tr>
<tr><td>*text</td><td>*access</td><td>*healthcare</td></tr>
<tr><td>text</td><td>access</td><td>healthcare</td></tr>
<tr><td>*mobile</td><td>*implementation</td><td></td></tr>
<tr><td>mobile</td><td>implementation</td><td></td></tr>
<tr><td>*alcohol</td><td>*american</td><td></td></tr>
<tr><td>alcohol</td><td>american</td><td></td></tr>
<tr><td>*diabetes</td><td>*body-image</td><td></td></tr>
<tr><td>diabetes | mellitus</td><td>body-image</td><td></td></tr>
<tr><td>*risk_communication</td><td>*challenge</td><td></td></tr>
<tr><td>risk_communication</td><td>challenge</td><td></td></tr>
</table>

Table S3: Full matrix of factor loadings.

		Component											
Construct	Factor	1	2	3	4	5	6	7	8	9	10	11	12
diet	1	0.922											
intervention	1	0.908											
physical_activity	1	0.889											
obesity	1	0.877				0.35							
nutrition	1	0.865											
prevention	1	0.841											
adult	1	0.833											
overweight	1	0.824											
program	1	0.799											
association	1	0.792							0.306				
fruit	1	0.783											
eating	1	0.781											
weight-loss	1	0.779											

healthy	1	0.771										
weight	1	0.771										
behavior_change	1	0.759										0.316
survey	1	0.756										
young_adult	1	0.748										
trial	1	0.742										
child	1	0.741			0.596							
control	1	0.733										
vegetable	1	0.727										
weight-gain	1	0.716										
low-income	1	0.714										
feeding	1	0.714										
parent	1	0.713										
mother	1	0.707										
lifestyle	1	0.699										
habit	1	0.697										
consumption	1	0.689		0.427								
risk-factor	1	0.685										
intake	1	0.679										
disease	1	0.676										
school	1	0.675										
randomized_controlled-trial	1	0.674										0.444
ehealth	1	0.673										
pattern	1	0.669										
adolescent	1	0.669			0.576							
public_health	1	0.66	0.325									
peer	1	0.648										
self-efficacy	1	0.643										
status	1	0.639										
practice	1	0.625							0.307			-0.32
infant	1	0.613										
nutrition_education	1	0.607										
choice	1	0.6		0.318	0.339							
gender	1	0.598							0.366			
meal	1	0.594										
population	1	0.591										
qualitative_research	1	0.59										
social_marketing	1	0.59										
environment	1	0.585				0.403						
prevalence	1	0.58						0.546				
pregnancy	1	0.576										
validation	1	0.574						0.373				
metaanalysis	1	0.568			0.423							
fast-food	1	0.567			0.55							
energy	1	0.566			0.364							
united-state	1	0.565										
american	1	0.565										
cooking	1	0.564									0.33	
nutritional	1	0.563										
family	1	0.555										
index	1	0.551									-0.31	
recipe	1	0.545										
sugar	1	0.543			0.477							
support	1	0.54										0.314
loss	1	0.535										0.313
group	1	0.523										
exercise	1	0.523										
education	1	0.52	0.313								0.407	
taste	1	0.52										
body-mass	1	0.518									-<br>0.329	
adherence	1	0.518										
barrier	1	0.516									0.326	
cancer	1	0.512										
student	1	0.511									0.325	
emerge	1	0.511										
young	1	0.501		0.338								
efficacy	1	0.499										
promotion	1	0.496			0.332							
policy	1	0.493			0.419							

recommendation	1	0.483										
healthcare	1	0.47										
home	1	0.469										0.308
life	1	0.469										
qualitative	1	0.466										0.355
time	1	0.466										
trend	1	0.463				0.389						
drinking	1	0.455				0.407						
research	1	0.451										
validity	1	0.431				0.377			0.304			
diabetes	1	0.42										
outcome	1	0.411										
focus	1	0.409										
assessment	1	0.408										
cognitive	1	0.404										
store	1	0.395										
design	1	0.395										
implementation	1	0.392						0.367				
inequality	1	0.391										
study	1	0.391										
stigma	1	0.381										
method	1	0.377										0.312
awareness	1	0.365										
people	1	0.338										
vegan_vegetarian	1	0.337										
evaluation	1	0.336										
factor	1	0.335										
process	1	0.327		0.308								
availability	1	0.323										
age	1	0.321										
bias	1	0.316										
label												
disparity												
natural												
individual												
photography												
university												
hotel	2		0.868									
online_reviews	2		0.868									
word-of-mouth	2		0.862									
review	2		0.845									
performance	2		0.836				0.317					
satisfaction	2		0.831		0.307							
restaurant	2		0.814									
service	2		0.808									
hospitality	2		0.8									
customer_satisfaction	2		0.799									
customer	2		0.793									
experience	2		0.751								0.452	
quality	2		0.74		0.354							
tourism	2		0.74								0.523	
ewom	2		0.737		0.306							
loyalty	2		0.727									
user-generated_content	2		0.727									
attribute	2		0.718									
tripadvisor	2		0.696									
impact	2		0.693	0.307		0.382						
antecedent	2		0.684									
electronic	2		0.649									
perceive	2		0.612		0.417							
brand	2		0.61			0.445						
sale	2		0.593									
value	2		0.58		0.481							
product	2		0.58		0.536							
motivation	2		0.562									
content	2		0.555									
word	2		0.537									
online	2		0.534	0.506								
big_data	2		0.533					0.483				
model	2		0.531	0.435	0.457							

moderate	2		0.531		0.399								
management	2		0.526	0.432			0.465						
role	2		0.505		0.432								
mouth	2		0.49										
industry	2		0.483				0.341						
sector	2		0.482		0.303								
corporate	2		0.471										
engagement	2		0.464	0.381									
decision	2		0.462		0.38								
involvement	2		0.46		0.34								
shopping	2		0.456										
orientation	2		0.456										
recovery	2		0.449										
user	2		0.444		0.312								
generation	2		0.44		0.414								
co-creation	2		0.424				0.349						
critical	2		0.415										
strategy	2	0.389	0.414			0.301	0.342						
development	2		0.396				0.349						
relationship	2		0.396		0.308								
new	2		0.389										
social-responsibility	2		0.367								-0.306		
corporate_social_responsibility	2		0.363										
facebook	2	0.308	0.339	0.327		0.301							
negative	2	0.325	0.326										
visual	2		0.316										
south													
virtual													
work													
food_safety	3			0.827									
risk_communication	3			0.788									
crisis	3			0.787									
amplification	3			0.76									
communication	3			0.73									
risk_perception	3			0.721	0.319								
public	3			0.709									
risk	3	0.441		0.704									
safety	3			0.689									
food_risk	3			0.673									
news	3			0.661						0.383			
coverage	3			0.641									
issue	3			0.632									
falsehood	3			0.6									
credibility	3		0.454	0.593	0.364								
modify	3			0.589									
trust	3		0.549	0.588	0.421								
source	3			0.585									
monitoring	3			0.563									
media	3		0.311	0.559		0.412				0.4			
science	3			0.558			0.392			0.312			
benefit	3			0.548									
knowledge	3	0.45		0.513									
fear	3			0.513									
perception	3		0.453	0.499	0.345						0.32		
internet	3	0.394		0.488						0.352			
traditional	3			0.47									
mass	3			0.469									
reputation	3		0.31	0.458									
governance	3			0.455			0.36						
genetically	3			0.449									
technology	3		0.31	0.441			0.324						0.363
frame	3			0.411						0.378			
framework	3			0.407									
emotion	3			0.404									
seek	3			0.4	0.322								
infection	3	0.358		0.399									
gratification	3			0.395									
illness	3			0.394									
web	3		0.334	0.393									

message	3	0.321		0.366							
foodborne	3			0.355							
perspective	3			0.345			0.343				
allergy	3			0.326							
tool	3			0.314							
unite											
organic	4				0.765						
purchase	4		0.406		0.757						
planned_behavior	4				0.755						
buying	4				0.722						
attitude	4			0.336	0.707						
intention	4		0.591		0.687						
food-intake	4				0.647						
belief	4				0.646						
acceptance	4		0.332	0.324	0.634						
theory	4	0.3			0.622						
consumer_behavior	4		0.343		0.586						
green	4				0.578		0.453				
determinant	4	0.314			0.522						
equation	4				0.509						
structural	4				0.505						0.326
norm	4				0.503						
social	4	0.402		0.398	0.472					0.329	
commerce	4		0.389		0.467						
willingness	4				0.439						
fit	4				0.437						
behavioral	4		0.384		0.429						
concern	4			0.345	0.413						
china	4			0.312	0.405						
meat	4				0.404		0.303				
opinion	4			0.356	0.402			0.32			
influence	4	0.343			0.384						
decision-making	4		0.365		0.377						
sharing	4		0.349		0.357		0.333				
personal	4				0.352					0.306	
action	4				0.319						
interaction											
advertising	5					0.915					
marketing	5					0.857					
unhealthy_food	5					0.829					
television	5	0.304				0.815					
exposure	5	0.36				0.81					
advergame	5					0.797					
beverage	5	0.382				0.775					
celebrity	5					0.757					
youtube	5					0.755					
endorsement	5					0.737					
influencer	5					0.697					
cue	5					0.638					
literacy	5	0.326				0.595					
alcohol	5	0.321				0.56					
digital	5					0.54				0.342	
response	5				0.308	0.526					
preference	5	0.317			0.356	0.484					
australium	5	0.381				0.473					
identification	5					0.444					
smoking	5	0.369				0.408					
self-management	5	0.382				0.391					
popularity	5		0.32			0.379					
power	5					0.358					
campaign	5	0.318				0.333					
attention											
sustainability	6		0.313		0.347		0.727				
agriculture	6						0.695				
innovation	6						0.684				
system	6						0.672				
network	6						0.666	0.32			
economy	6						0.639				
chain	6						0.636				
alternative	6						0.63				

supply	6						0.622					
farmer	6						0.6					
rural	6						0.597					
climate	6						0.531					
waste	6				0.374		0.522					
local	6		0.359				0.521				0.33	
space	6						0.516			0.368		
entrepreneurship	6						0.511					
movement	6						0.506					
community	6		0.32				0.494					
urban	6						0.467					
trade	6						0.454					
market	6		0.329				0.445					
resource	6						0.438					
dynamics	6						0.419					
organization	6			0.327			0.416					
justice	6						0.416					
city	6						0.411					
activism	6			0.37			0.404					
reduction	6						0.403					
business	6		0.368				0.395					
access	6						0.381					
challenge	6			0.346			0.378					
change	6	0.304					0.355					
citizen	6						0.326					
social_media_marketing	6						0.312					
global												
food_security												
state												
resistance												
acculturation												
sentiment_analysis	7		0.374					0.74				
tweet	7							0.713				
halal	7							0.704				
datum	7							0.694				
detection	7							0.686				
twitter	7			0.388				0.684				
topic	7							0.679				
mining	7		0.316					0.641				
classification	7							0.612				
sentiment	7		0.467					0.61				
analytic	7		0.458					0.591				
text	7							0.57				
modeling	7							0.553				
data_mining	7							0.527				
muslim	7							0.516				
geography	7						0.334	0.512		0.313		
analysis	7		0.475					0.5				
spatial	7							0.482				
surveillance	7							0.429				0.333
machine	7		0.317					0.411				
social_network	7		0.324					0.394		0.346		
learning	7		0.351					0.384				
world	7							0.333				
disaster	7							0.332				
sport												
depression	8								0.793			
anxiety	8								0.749			
addiction	8								0.72			
disorder	8	0.308							0.686			
symptom	8								0.638			
distress	8								0.549			
psychological	8		0.345						0.516			
stress	8	0.31							0.511			
well-being	8	0.331							0.507			
personality	8				0.331				0.505			
sensitivity	8								0.483			
scale	8		0.418		0.369				0.465			
use	8			0.423					0.453			
mental	8	0.408							0.449			

covid-19	8								0.415				
event	8								0.364				
ethnic													
discourse	9									0.557			
blog	9	0.386		0.308						0.522			
body-image	9									0.486			
eating_disorder	9								0.428	0.476			
body	9	0.421								0.466			
politics	9			0.302			0.438			0.466			
narrative	9									0.463			
anorexia	9									0.445			
site	9		0.315							0.393			
nervosa	9									0.377			
ethnography	9									0.375			
culture	9									0.366	0.33		
participation	9						0.312			0.363			
platform	9									0.354			
identity	9									0.353			
ethic	9									0.347			
street	9									0.308			
self	9									0.305			
need													
poverty													
destination	10		0.527								0.645		
authenticity	10										0.64		
place	10		0.314				0.347				0.586		
culinary	10		0.333								0.583		
image	10		0.554								0.562		
travel	10		0.42								0.525		
wine	10		0.357								0.504		
content_analysis	10		0.389								0.486		
gastronomy	10		0.441								0.467		
instagram	10										0.318		
country													
netnography													
video													
drug	11											0.486	
medical	11											0.432	
patient	11											0.413	
adverse	11											0.368	
build	11											-0.342	
fda	11											0.34	
neighborhood	11	0.321						0.313				-0.339	
prescription	11											0.334	
warning	11											0.331	
effect	11											0.314	
care													
application	12												0.621
mobile	12	0.383											0.619
(smart)phone	12	0.376											0.556
app	12	0.326											0.515
adoption	12		0.314	0.33									0.358

Extraction Method: Principal Component Analysis.

Rotation Method: Varimax with Kaiser Normalization
Rotation converged in 15
iterations.
